# Exploring AuRh Nanoalloys: A Computational Perspective on the Formation and Physical Properties

**DOI:** 10.1002/cphc.202200035

**Published:** 2022-03-14

**Authors:** Mirko Vanzan, Robert M. Jones, Stefano Corni, Roberto D'Agosta, Francesca Baletto

**Affiliations:** ^1^ Department of Chemical Sciences University of Padova Via Marzolo 1 35131 Padova Italy; ^2^ Department of Physics King's College London Strand London WC2R 2LS UK; ^3^ CNR Institute of Nanoscience Via Campi 213/A 41125 Modena Italy; ^4^ Department of Polymers and Advanced Materials: Physics, Chemistry and Technology (PMAS) Universidad del País Vasco UPV/EHU Avenida de Tolosa 72 20018 San Sebastián Spain; ^5^ IKERBASQUE Basque Foundation for Science Plaza de Euskadi 5 48009 Bilbao Spain; ^6^ Department of Physics University of Milano Via Celoria 16 20133 Milano Italy

**Keywords:** nanoclusters, nanoalloys, AuRh, synthesis, density functional calculations, multiscale modelling

## Abstract

We studied the formation of AuRh nanoalloys (between 20–150 atoms) in the gas phase by means of Molecular Dynamics (MD) calculations, exploring three possible formation processes: one‐by‐one growth, coalescence, and nanodroplets annealing. As a general trend, we recover a predominance of Rh@Au core‐shell ordering over other chemical configurations. We identify new structural motifs with enhanced thermal stabilities. The physical features of those selected systems were studied at the Density Functional Theory (DFT) level, revealing profound correlations between the nanoalloys morphology and properties. Surprisingly, the arrangement of the inner Rh core seems to play a dominant role on nanoclusters’ physical features like the HOMO‐LUMO gap and magnetic moment. Strong charge separations are recovered within the nanoalloys suggesting the existence of charge‐transfer transitions.

## Graphical abstract

**The synthesis and physical properties of AuRh nanoalloys**, with size range between 20–150 atoms, are studied by means of molecular dynamics and density functional theory calculations. The best synthetic route towards stable nanosystems is reported along with their physical features, which show unique characteristics that have active potential in photocatalytic applications.

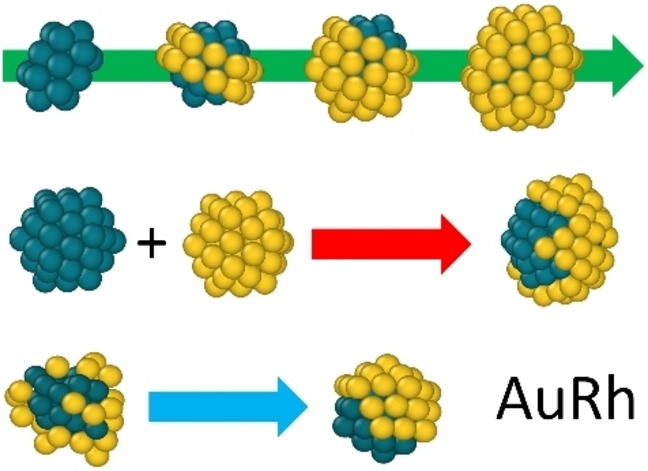

## Introduction

Most of the nowadays chemical production is based on reactions catalysed by transition metals and their alloys. These catalysts usually offer high activities and recyclability, but suffer from poor selectivity and, usually, require a high amount of energy to work properly.^[1]^ A possible way to overcome these problems is by considering nanoalloys. With their reduced dimensions (less than 2 nm) and alloying metals with different properties, such ultra‐small systems are characterised by strong quantum confinement effects. This is the reason for their unique properties in terms of opto‐electronic features, magnetism and catalytic capabilities.[^2^, ^3^, ^4^, ^5^, ^6^] The adaptability and the catalytic efficiency of these nanosystems can be further enhanced by alloying two or more metallic species, which structural and electronic properties are significantly different from those of the individual constituents.[^7^, ^8^, ^9^, ^10^, ^11^, ^12^] Furthermore, at the nanoscale it is possible to alloy bulk‐immiscible elements giving rise to new unexplored compounds, as in the case of gold‐rhodium nanoalloys. According to literature, gold and rhodium are almost immiscible in bulk: the dissolution of the metals is below 1 % even when the temperature reaches 1200 K.[^13^, ^14^] This is likely related to the intensity of metal‐metal mutual interactions: homopolar interactions, especially for Au,^[15]^ are stronger than heteropolar Au−Rh interactions. This favours metals segregation and prevents effective atomic mixing, even in the liquid phase. However, at the nanoscale Au and Rh form hybrid structures which were proved to be very effective in catalysing light‐induced reactions such as hydrogen generation from water,^[16]^ oxygen evolution,^[17]^ H_2_O_2_ synthesis^[18]^ and NO reduction in presence of carbon monoxide.[^19^, ^20^] AuRh nanoalloys also show superb catalytic activity towards carbon‐based compounds, as in the case of tetralin hydroconversion with H_2_S,^[21]^ alkenes reduction with gaseous hydrogen^[22]^ and CO oxidation.^[23]^ Finally, it was recently shown that such systems have strong bactericide action towards drug‐resistant bacteria.^[24]^ Despite the extraordinary performances and adaptability, AuRh alloys have been rarely investigated to date, mostly because batch‐synthesized AuRh particles are mildly stable and tend to dissociate.^[25]^ Most of the available data are therefore related to surface‐supported systems, using as supporting materials TiO_2_ or Al_2_O_3_. However, it was shown that such an experimental setting induces phase segregation, affecting the catalytic potential that remains largely unexpressed.[^21^, ^26^] Thus, finding a feasible synthetic way to obtain stable AuRh alloys that do not segregate is a crucial point that must be addressed.

To date, the computational efforts spent on the study of this nanoalloy mainly focus on the characterisation of the systems, barely accounting for its dynamics. Several studies indicate how the most stable configuration for the isolated nanoalloy seems to be the one with the rhodium encapsulated inside gold, forming a core‐shell structure, consistent with arguments pertaining to the relative surface energy of the two components.^[27]^ Interestingly, this feature was recovered either in small aggregates (few dozens of atoms) or in large systems (more than 10000 atoms), using both ab‐initio and classical atomistic approaches.[^28^, ^29^, ^30^, ^31^] Despite the evidence, there is no general consensus among the theoretical and experimental communities on which are the more favorable geometrical arrangements, how these depend on variables as the relative metal composition or the operating temperature[^32^, ^33^, ^34^] and what is the relation between the structure and the photocatalytic properties. Indeed, very recent studies seem to undermine the net stability of core‐shell pattern compared to others configurations, suggesting a hybrid mixed‐Janus motif as the most favorable arrangement.[^35^, ^36^] While in parallel, other investigations started to shed light on the dynamics behind the synthetic process of these nanoalloys in some particular cases.^[37]^

To date, what appears to be certain by mixing Au and Rh is that the shapes and properties strongly deviate from the ones of pure metal systems and this mismatch can be recovered even in aggregates composed of a few atoms.^[38]^ Moreover, it seems that in these aggregates an odd number of atoms stabilise the system and that the optimal Au : Rh ratio is around 50 %.^[39]^ This naturally prompts the question: do these pieces of evidence still apply to larger system sizes? Understanding the relative stabilities of the various isomers and homotopes^[40]^ as a function of their size and chemical composition is a fundamental step to deeply understand their catalytic activity. To do that, it is necessary to study the formation process of the alloys using atomistic resolution and correlate the geometrical properties of the obtained structures with their physical features. We therefore conducted a computational study to investigate the synthesis and the physical properties of AuRh nanoalloys in the subnanometer regime, between 20–150 atoms, by means of a multi‐scale approach. We combine classical Molecular Dynamics (MD) and Density Functional Theory (DFT) simulations. The former method was applied to explore three different synthetic routes commonly adopted in the nanoalloys gas‐phase synthesis, namely the one‐by‐one growth over a small metallic seed, the collision between nanostructures with different dimensions, and the annealing of liquid nanodroplets.^[41]^ By analysing the evolution of the energetics along the simulations, we could identify the most favourable nanoalloy morphology and their likelihood of being formed. The latter technique was further exploited to study their physical properties and their correlation with the nanoalloys shapes, obtaining interesting connections between the nanocluster structures and features.

## Results and Discussion

**Figure 1:**
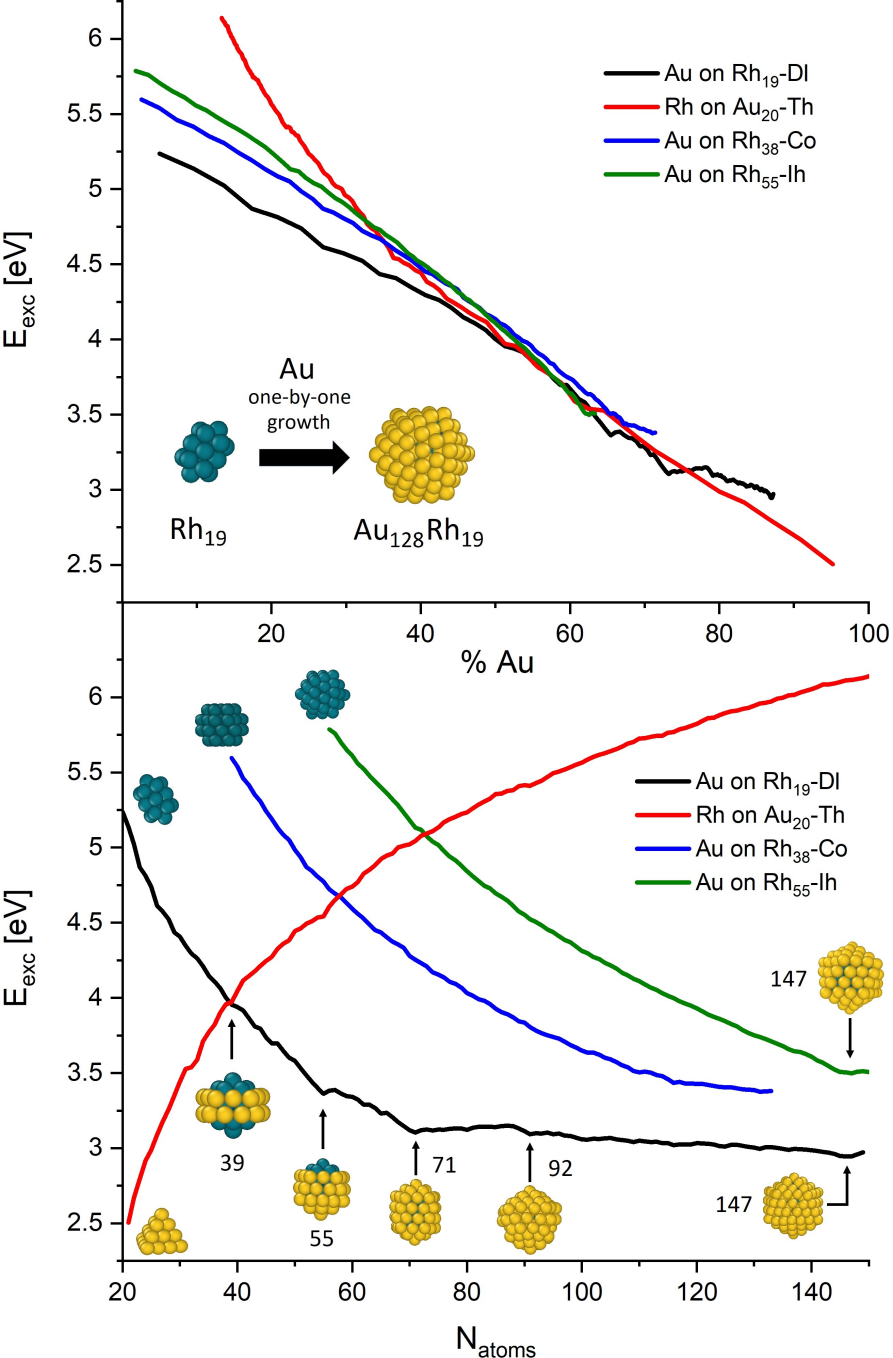
Excess energy as a function of the composition (upper panel) and total number of atoms (bottom panel) for the AuRh formation through one‐by‐one growth in gas phase. Inset images at the beginning of the curves represent the starting metal seeds, while the others are schematic representations of notable structures. Blueish and yellow balls represent Rh and Au atoms respectively. Plotted curves are averaged over 10 trajectories, error bars are omitted, curves are given within ±0.1 eV.

The first explored synthetic route was the one‐by‐one growth in the gas phase. Here, a nanocluster of a certain species (Au or Rh) acts as a nucleation site for the nanoalloy growth which is performed by subsequent deposition of atoms of the other species. We explored the growth of Au over three different Rh seeds, namely the Rh_19_ double‐icosahedron (Rh_19_‐DI), Rh_38_ truncated cuboctahedron (Rh_38_−Co), and Rh_55_ icosahedron (Rh_55_−Ih), while the growth of Rh atoms over gold was performed only for a single seed, i. e. the Au_20_ tetrahedron seed (Au_20_−Th). Graphical representations of these nanoclusters, as well as the excess energy trend as a function of the alloy composition and total number of atoms are collected in Figure 1. The excess energy is defined as the energy loss with respect the same number of atoms in the bulk, divided by an estimate of the number of atoms at the surface (see Equation 1 in the Computational Methods section). The choice of such particular seeds come from what is already known about the structure of small metal clusters that tend to generate compact aggregates with well‐defined geometries. 38‐Co and the 55‐Ih are configurations commonly assumed by many metal aggregates,^[32]^ while the Au_20_−Th and Rh_19_−DI are known stable structures for these two particular metal species.[^42^, ^43^] As visible from the excess energy plots, the thermodynamic and the stability of the systems strongly depend on the size and composition. In particular, it appears that the larger the amount of gold the lower the excess energy of the alloy. This is because the bulk cohesive energy, which directly influences the excess energy as reported in Equation (1), is remarkably lower for gold compared to rhodium (c.a. 3.8 eV vs 5.9 eV)^[44]^ and therefore larger Au : Rh ratios are characterised by smaller excess energies. The latter argument also explains why the growth of Rh over Au_20_−Th (red line) seems to follow an opposite trend in the bottom panel of Figure 1. Notably, each line in the graphs presents a non‐monotonic behavior and local energy minima. This is particularly evident in the case of Au deposition over Rh_19_‐DI (black line) where 5 local minima can be identified. These points correspond to the 5 morphologies, depicted at the bottom panel of Figure 1. Those morphologies have an Rh_19_ core enclosed in a gold‐shell which covering extension and motif depend on the number of Au‐atoms. Among these 5 structures, the ones with lower number of atoms are worth of particular interest as they present very unusual and exotic arrangements. The 39 atoms Au_20_Rh_19_ structure is composed by an inner Rh_19_ core surrounded by an atomic gold stripe; the 55 icosahedral Au_36_Rh_19_ system presents a ball‐cup arrangement where an Rh tip emerges from the inner part of the icosahedron and the 71 atom Au_52_Rh_19_ which shows a chiral core‐shell motif. For the sake of brevity from now on these clusters will be named 39‐GS (gold stripe), 55‐BC (ball‐cup) and 71‐CM (chiral motif).

**Figure 2:**
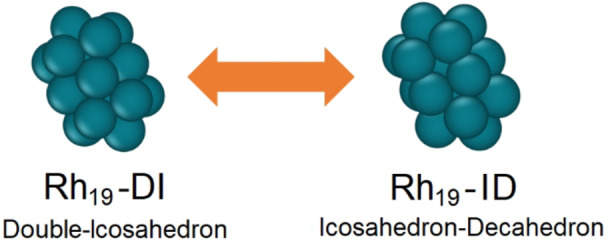
Graphical representation of the two configurations assumed by the Rh_19_ seed along the nanoalloys growth.

At this stage of the investigation, it is worth noticing that the configuration of the Rh_19_ seed seems to play a major role in determining the formation process of those structures. While in the case of 71‐CM, the seed still shows a double‐icosahedron symmetry, in the case of 39‐GS and 55‐BC, the Rh_19_ seed presents a fused icosahedron‐decahedron geometry, as visible from Figure 2. We never observed the Rh‐seed assuming a double‐decahedron, but the Ih−Dh is similar to what recently found in ab‐initio MD of Al‐nanoalloys.^[45]^ This indicates that geometrical modification can occur within the nanoalloy cores during the formation process and that these changes directly influence the stability of the whole nanoalloy and drive its growth. We will refer to this seed configuration as Rh_19_−ID. The role the seed geometry has on the whole structure stability, together with a thorough analysis of the mentioned notable structures is given in the following section, where DFT calculations results will be presented and discussed.

The two morphologies corresponding to the other excess energy minima in the one‐by‐one deposition of Au over Rh_19_ (black curve in Figure 1) corresponds to the 92 atoms Au_73_Rh_19_ and the 147 atoms Au_138_Rh_19_ respectively. These structures present more predictable and less unique shapes compared to the previous ones, since they possess complete (Au_138_Rh_19_) or incomplete (Au_73_Rh_19_) icosahedral geometries.^[46]^ Therefore, despite these structures are marked by relative low‐energy geometries, we did not include them in our examinations. Our analysis is indeed dedicated to the sole structures which, due to their exotic shapes, could present notable and unique physical and catalytical properties. The discarded nanoclusters will be however considered in the simulations investigating nanodroplets annealing, as we will discuss later.

Focusing on the growth over the other Rh seeds (Rh_38_‐Co and Rh_55_‐Ih), the excess energy trend does not reveal any particular structure corresponding to a given nanocluster size or composition, except for the icosahedral Au_92_Rh_55_ 147 atoms nanocluster, coming from the growth of Au over Rh_55_−Ih which is stable due to the geometrical closure of the shell enveloping the inner seed. Lastly, the deposition of Rh atoms over a gold seed does not produce any notable structures. The mild variation of the excess energy trend in Figure 1 (red line) refers to geometries in which the gold atoms of the seed migrate toward the external surface of the nanocluster, locally smoothening the monotonical growth of the excess energy. Obtained Rh‐rich systems do not produce high symmetry structures, preferring to form aggregates reminding the FCC structures of bulk rhodium. The remarkable propensity of Au atoms to migrate towards the surface notwithstanding its small diffusion coefficients^[47]^ (it was estimated in the order of 10^−17^ cm^2^/s) suggests a strong tendency to form segregated phases with Rh preferably confined within a gold shell, in line with what observed in the previously discussed simulations where the seed was made of Rh atoms. Despite the general consensus in reputing the Rh@Au core‐shell arrangement as the most stable for AuRh arising from computational inspections,[^28^, ^29^, ^30^, ^31^, ^48^] this is the first time the arising of this chemical order is directly observed with atomistic accuracy in such small nanoclusters through MD simulations. This tendency to form alloys with this specific chemical ordering can be justified by the higher cohesive of Rh atoms compared to Au, which naturally try to rearrange in order to minimise the exposed surface and saturate all pending bonds. Having in mind photocatalytic applications where the strong plasmonic properties of gold are combined with the catalytic capabilities of rhodium, this phase segregation could heavily affect the device performances since no Rh atoms are available to bond molecular species on the surface of the nanosystems, and no plasmonic Au nucleus is easily forming. Thus, searching for strategies that promote synthesis of alloys rich in surface Rh content is currently a major challenge that must be faced. Simply increasing the Rh content of the alloy is not a viable solution, since the disordered rearrangement of the gold atoms on the surface would dramatically affect the optical properties of the system, which would be in turn dominated by the poor optical response of rhodium thus losing the main driver for the photocatalysis.^[49]^ Within this framework, the 39‐GS and 55‐BC systems could present a concrete possibility for catalytic applications since they expose Rh atoms on the surface while maintaining well‐defined structures and thus specific and well distinguishable electronic transitions. For photocatalytic purposes, 71‐CM could also present as a valuable candidate due to its chiral superficial texture that could bind molecules with a chiral configuration, even though the shell is made of gold atoms.

**Figure 3:**
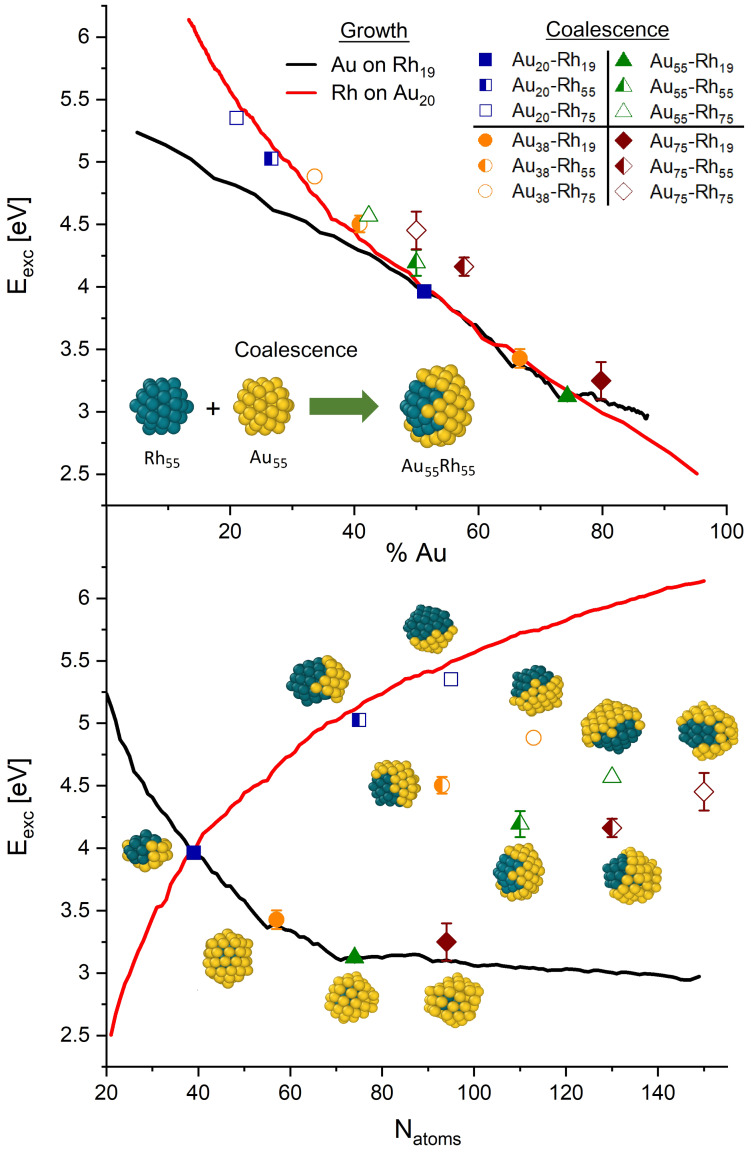
Excess energy as a function of the chemical composition (upper panel) and the total number of atoms (bottom panel) for the AuRh formation through coalescence (coloured symbols). For the sake of comparison, the graphs include the excess energy trends of the one‐by‐one growth in the case of Au_20_−Th and Rh_19_−DI seeds. Inset images represent the alloys recovered from the coalescence simulations. Error bars are the standard deviation of the excess energy calculated over 10 trajectories.

The second explored synthetic route was the coalescence between pure nanoclusters in the gas phase. The complete list of structures we chose for these simulations is available in the Computational methods section, while the excess energy of the obtained alloys as a function of composition and dimensions are given in Figure 3. As expected, the stability of the coalesced nanoclusters directly depends on the ratio between the two metals: the higher the Au : Rh ratio, the lower the excess energy. Notably, regardless of the dimension of the gold counterpart, the coalesced nanoclusters coming from the collision of Rh_19_ have the lowest excess energies and closely follow the trend recovered in the case of the one‐by‐one growth, included in Figure 3 as a reference to evaluate the coalescence efficiency. We suspect that, due to its small dimension, the Rh nanocluster can be fully enveloped within a gold shell and this rapidly occurs to minimise the surface energy, similar to what is observed in the one‐by‐one growth. Minor differences aside, the structures obtained in this case have morphologies which are surprisingly similar to the ones of the gold deposition over Rh_19_ seed. An interesting exception is observed in the case of the coalescence between Au_20_ and Rh_19_ which produce a nanocluster where the Rh kernel is partially covered by Au atoms, in a covering motif quite different from the one of the 39‐GS case obtained during the deposition.

Despite the different structural arrangements, isomers obtained through these two synthetic approaches have sufficiently similar excess energy, indicating the same relative stability compared to other possible shapes. This suggests that, although coalescence can produce structures energetically identical to the one of the growths over seeds in some specific cases, this technique can be used to synthesise isomers of the same nanocluster whose properties could be sensibly different from its one‐by‐one growth counterpart. These considerations are however valid only in some specific cases. As observable from Figure 3, most of the coalesced alloys strongly deviate from the ones of the metal deposition over a seed, either considering the growth over Au_20_‐Th and Rh_19_‐DI (red and black lines in Figure 3). Most of the simulations produce non‐symmetric shapes which often are energetically higher than those obtained from the one‐by‐one growth at the same Au : Rh compositions (see for example the results concerning the coalescence of Au_75_). In general, they have energies higher than the ones obtainable through metal deposition and nucleation on a seed. At finite temperatures, it is likely that coalesced morphologies will evolve to those shapes obtained from the one‐by‐one growth. We do not consider asymmetric morphologies for further analysis. Before proceeding with the presentation of the results concerning the last explored synthetic procedure, it is interesting to highlight the spontaneous propensity to generate Rh@Au core‐shell nanoclusters where the Au atoms tend to cover the surface of the Rh counterpart as is noticeable in the inset images reported in Figure 3, confirming the natural predisposition observed in the previously analysed synthetic route.

Finally, we consider our tertiary formation process for AuRh nanoalloys is the annealing of small nanodroplets. In these simulations, some of the tested systems were collected from the trajectories of the processes already discussed, while others were specifically built for this purpose. A detailed list of the tested systems is available in the Computational method section. Compared to the previous synthetic approaches, this process allows for the formation of the thermodynamically stable isomer for a certain nanocluster and this highlight which structures are more likely to be obtained in an opportunely designed experimental setup. Indeed, our simulations focus on the formation of individual nanoclusters but neglect the coexistence or the assembly of several nanoparticles at finite temperatures. Our calculations should be treated as a reference for gas‐phase experiments where the concentration of nanoalloys is low enough to prevent sensible clusters interactions.[^50^, ^51^] Furthermore, the size differences considered here are so small that one can neglect Ostwald ripening.^[52]^

**Figure 4:**
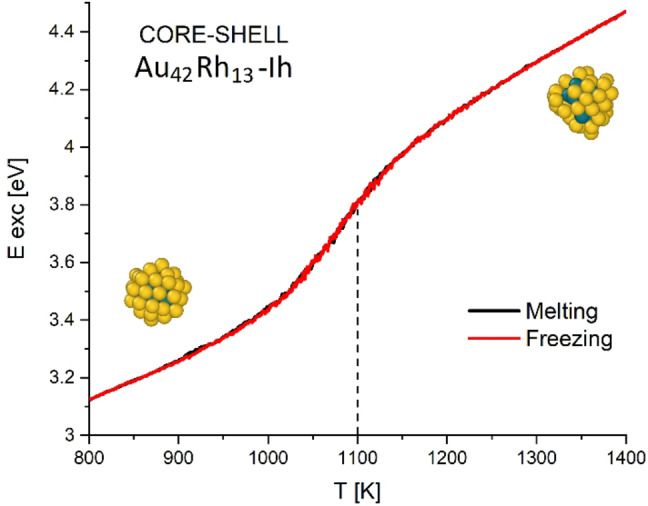
Caloric curves for core‐shell Au_42_Rh_13_ nanocluster for both melting and freezing processes. Inset images represent the alloy conformation at 800 K and at 1400 K.

We calculate the meting temperature (T_m_) of the investigated alloys, since the solid‐liquid phase transition can be directly observed from the trend of the excess energy as a function of the temperature in both melting and subsequent freezing processes. An example of this plot is given in Figure 4 where the excess energy for the 55 atoms core‐shell Au_42_Rh_13_ icosahedral nanocluster is given as a function of temperature, in both processes. In this case, the systems present a phase transition that begins at 1000 K and terminates around 1200 K, leading to a liquid nanodroplet. We define the T_m_ of the nanocluster as the inflection point of the functions which in this case falls around 1100 K. The overlap of the two lines referring to the melting and freezing processes indicates that the transition is thermodynamically reversible and that the geometries recovered from the solidification, i. e. the thermodynamically stable isomer of the alloy, coincide with the nanocluster initially melted.

As a general trend, we find that all investigated nanoalloys present excess energy trends similar to the ones of Figure 3. However, there were specific cases where the plots show discontinuities and non‐monotonical trends for the excess energy dependence on the temperature. This is especially the case for the systems we built to study the stabilities of Janus, inverse core‐shell, and randomly mixed chemical ordering.

**Figure 5:**
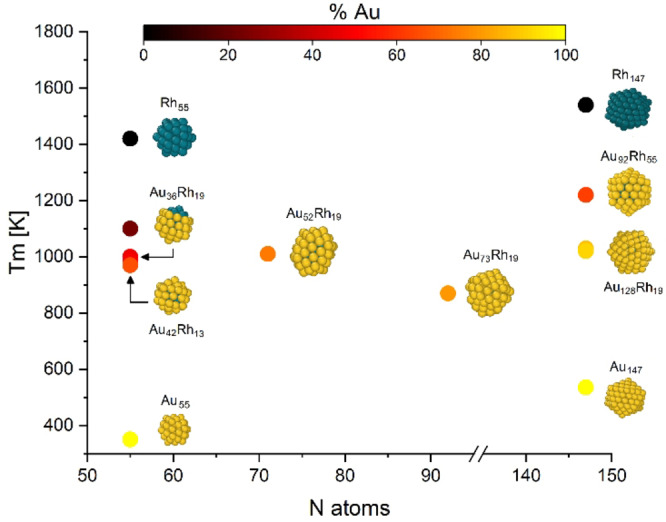
Melting temperatures of the investigated nanoalloys, as a function of size and metal content. The images are graphical representations of the particular systems. T_m_ are given within a confidence interval error of ±50 K.

The uncommon tendencies of their excess energy, which are available as Supporting Information, indicate large instabilities and testify to the presence of phase transition toward the Rh@Au core‐shell chemical order even below 300 K, thus highlighting the high instabilities of these arrangement compared to the core‐shell one. Apart from these cases, most of the investigated nanosystems present annealing patterns that confirm the thermodynamical stability of the structures annealed, presenting no mismatches between the excess energy trends for the melting and freezing simulations and well‐defined T_m_. Dynamically, it appears that the solid‐liquid transition is generally driven by the integrity of the gold shell. In the case of the melting process, as the temperature approaches T_m_, the atoms constituting the Au shell start to vibrate and diffuse, losing a coherent motif while the inner Rh seed remains compact. As the temperature exceeds T_m_, the rhodium kernel starts to lose its structure and gradually melts. This phenomenon, always observed in our systems, can be interpreted on the basis of the following considerations. First, T_m_ for gold is sensibly lower than the one of rhodium (1337 K vs 2237 K in the bulk phase^[53]^) indicating a weaker temperature stability for gold structures. Secondly, the rhodium core is usually enveloped within the gold shell in a compact shape where the atom coordination numbers are generally higher than the ones of the gold atoms constituting the shell. Therefore, the binding energy per atom is higher for Rh than Au. This does not mean the Rh kernel does not play an active role in the process. In the case of 71‐CM, for example, the rotational motion of Rh_19_‐DI seed is activated by the temperature, allowing a continuous swap between Rh_19_‐DI and Rh_19_‐ID shapes, whose graphical models are presented in Figure 2. A plot showing the T_m_ as a function of the cluster sizes and metal contents for the investigated nanoalloys is available in Figure 5.

Pure Au and Rh icosahedral structures made of 55 and 147 atoms were included in our analysis as a benchmark to evaluate the features of the nanoalloys. As expected, the T_m_ of systems owning the same number of atoms (e. g. when N=55, 147 in Figure 5) is between the values identified for the pure nanoclusters. This demonstrated that T_m_ is a multifactorial function that depends on both system size and composition. Such dependencies are however not so trivial as could be thought in the first place.

It is indeed surprising how the T_m_ of 71‐CM is 140 K higher than the one of the 92 atoms structure Au_73_Rh_19_. Such a temperature difference is too large to be justified on the basis of the sole difference in metal content which is indeed quite moderate (73 % of Au content for 71‐CM vs 79 % in the case of Au_73_Rh_19_) and anyway is not coherent with the fact that the 147 atoms Au_128_Rh_19_ present a higher T_m_ compared to Au_73_Rh_19_. Furthermore, all those structures arise from the same synthesis, and thus this peculiar T_m_ behaviour cannot be related to differences in chemical ordering or nanoclusters inner morphology. The explanation of this phenomenon must lie elsewhere. Thinking about the relative structures stabilities it can be noticed that the 71‐CM and the Au_128_Rh_19_ nanoclusters possess remarkable energetic stabilities given by the geometrical closure of the gold shell surrounding the inner part of the system. Being stacked within a well‐defined texture, the gold atoms have a high kinetic diffusion barrier and texture is less affected by the enhanced thermal vibrations. Conversely, the superficial atoms of Au_73_Rh_19_ are not constrained within any particular motif and further suffer from low‐coordinated Au atoms, making them more sensitive to the temperature changes. Generally speaking, symmetric isomers are likely to show a higher melting point than less symmetrical structures, because of a lower contribution to the vibrational entropy.^[54]^ At least from a qualitative point of view, we can argue that the higher symmetry of 71‐CM compared to the 92‐atom structure should justify the observed T_m_ differences.

Another interesting aspect that emerges from the analysis of T_m_ is that all nanoalloys show remarkably high thermal stabilities. There are nanoalloys such as 55‐BC with remarkably high T_m_ (around 1000 K), which is an outstanding value for a 55 atom aggregate. The case of 147 atom structures is even more surprising. Even though some of them have a very low Rh content (around 13 % in the case of Au_128_Rh_19_), the T_m_ are still surprisingly large, being located around 1000 K. This indicates that even a small amount of Rh can heavily modify the physical properties of these systems and influence their thermal stabilities. This, in turn suggests that an Rh doping of gold structures could effectively increase the thermal stability of the system and extend the range of temperature in which gold‐nanostructured devices can be applied, potentially leading to important improvements in technologically relevant applications.

These latter observations, which are here based on a theoretical approach to the problem, follow what is experimentally known about the thermal stabilities of these nanoalloys. In a work from 2017, Shubin et al. studied the formation and stability of gold‐based nanoalloys exploring also the AuRh system.^[37]^ Despite the investigated sizes and compositions were slightly different from the ones of our nanosystems, their analysis reflects what we observed in our simulations. High Temperature Synchrotron X‐Ray Diffraction techniques revealed that AuRh nanoalloys have a remarkable thermal stability (even though smaller than the ones we observed) and the authors suggested this is due to the slow kinetics of rearrangements which require a large amount of thermal energy to be activated. Our simulations are compatible with the experimental observations on the impressive thermal stabilities and the high kinetic energy barriers, while highlighting the importance of the latter and its connection with the geometrical arrangements of the atoms. We indeed recover a non‐monotonic trend in the size dependence of T_m_ which we related to the structures of the alloys themselves and their structural closure.

To conclude this section on the results of MD simulations, we want to comment and integrate the synthetic mechanism Shubin et al. proposed in their work. From the data obtained through X‐Ray Diffraction, they proposed the synthesis of AuRh could occur through a *conversion chemistry* mechanism where Au seeds act as nucleation spots for Rh growth, which gradually migrates towards the inner part of the structure as the sizes increase.^[37]^

The experimental conditions indeed forced the synthesis to proceed that way and it was demonstrated to generate thermally stable nanoclusters. However, the melting temperature recovered by the authors are sensibly smaller compared to the ones we predict in our simulations, being around 700–800 K for nanoparticles with sizes around 5 nm. In our opinion this is due to the chosen synthetic procedure (Rh nucleation over Au seeds), which we demonstrated is not the most suitable for these kinds of nanoalloys, leading to relatively unstable systems. On the contrary, performing the growth the opposite way i. e., by depositing Au onto Rh seeds could bring us to structures with higher thermal stabilities, lower topological disorder and could lead to a more accurate atomic species distribution, selectively producing minimum energy alloy isomers.

In the second part of this work we deepen our insight on some specific AuRh structures and investigate their features by means of DFT calculations, using a plane‐waves basis and the PBE exchange‐correlation functional.^[55]^ Geometrical coordinates of the DFT‐relaxed nanoalloys are available in the Supporting Information. We focused our treatment on the formerly described 39‐GS, 55‐BC and 71‐CM nanoclusters since their exotic shapes together with their remarkably relative stability makes them good potential candidates for photocatalytic applications. Each of the mentioned cases was studied in two different shapes. As noticed in the one‐by‐one growth simulations, the geometry of the inner Rh seed seems pivotal to determine the structure and stability of the nanoalloys. We therefore investigated the mentioned alloys taking into account two different isomers, namely Rh_19_‐DI and Rh_19_‐ID. We furthermore extend the calculations on the pure forms of metal structures, i. e. on Rh_19_, Au_20_‐Th, Rh_55_‐Ih and Au_55_‐Ih. The data recovered from the DFT calculations are collected in Table 1. Starting from the systems energetics, the nanoclusters isomer which resulted energetically favoured from the finite‐temperature MD simulations still resulted the most favourable structures in the DFT simulations. This remains valid also for the MD stabilities calculated at T=0 K as we are going to discuss soon. The DFT estimated energetic trend is confirmed by the values of the bonding energy E_bond_, which estimates the nanoalloys stability with respect to the isolated atoms case (see Computational Methods for more details). Such values are always higher for the isomers recovered through MD (marked with * in Table 1) compared to the counterpart (e. g. for 39‐GS E_bond_ is larger for ID configuration, while for 71‐CM is larger in the case of DI arrangement). However, the relative stability between the two isomers is not constant and seems to increase as the gold shell covers the inner seed. The total energy separating the two isomers at the DFT level are 0.16 eV, 0.24 eV and 1.36 eV for 39‐GS, 55‐BC and 71‐CM nanostructures, respectively. We calculated the relative stabilities between isomers at the MD level, quenching the structures obtained during the dynamics. We obtained energy differences between DI and ID isomers of 0.66 eV, 0.80 eV and 1.08 eV for 39‐GS, 55‐BC and 71‐CM, respectively. Although the agreement with DFT is only qualitative as expected, the energy ordering is the same, suggesting with a larger gold envelop, it is less likely to rearrange its core. Energetically speaking, there is also a net energy gain in forming the alloy compared from the pure nanoclusters, as confirmed by the positive values of the mixing energy ϵ_mix_. This indicates that the production of those systems should be an exothermic process that occurs spontaneously and could in principle catalyse the production of isomers with similar energy, if the kinetic energy barrier for the isomer's transition is small enough.

**Table 1:** Data collected from DFT calculations performed on various pure and alloyed nanostructures. Here d indicates the average bond length between nearest neighbour for the couple of species indicate as subscript, E_bond_ and ϵ_mix_ are the bonding and mixing energy, M is the average magnetisation per atom and H−L indicate the energy of the HOMO‐LUMO gap. Error bars associated to d indicate the standard deviation of the sets of bonds in each cluster. * stands for the classically predicted lowest energy isomer.

Name	Shape	*d*_Au−Au_ [Å]	*d*_Rh−Rh_ [Å]	*d*_Au−Rh_ [Å]	E_bond_ [eV]	ϵ_mix_ [eV]	*M* [μ_B_]	H−L [eV]
39‐GS	DI	2.80±0.02	2.72±0.17	2.77±0.10	3.419	0.297	0.07	0.03
	ID*	2.88±0.07	2.71±0.07	2.82±0.07	3.423	0.255	0.64	0.14
55‐BC	DI	2.85±0.08	2.74±0.08	2.80±0.08	3.303	0.178	0.18	0.15
	ID*	2.89±0.04	2.69±0.05	2.81±0.04	3.307	0.146	0.32	0.16
71‐CM	DI*	2.87±0.04	2.71±0.06	2.80±0.05	3.213	0.186	0.18	0.11
	ID	2.85±0.04	2.70±0.06	2.79±0.05	3.194	0.181	0.22	0.08
Rh_19_	DI*	–	2.67±0.04	–	3.888	–	0.55	0.08
	ID	–	2.63±0.08	–	3.876	–	0.41	0.18
Au_20_	Th	2.81±0.10	–	–	2.360	–	0.00	1.79
Rh_55_	Ih	–	2.65±0.06	–	4.489	–	0.54	0.10
Au_55_	Ih	2.84±0.07	–	–	2.527	–	0.00	0.65

The geometrical analysis of the DFT‐relaxed nanoalloys reveals interesting trends for the nearest neighbour interatomic distances. At first, our analysis of the data demonstrates that as the size of the system increases, the average Au−Au bond length increases and approaches the bulk PBE estimated reference value of about 2.95 Å.^[56]^ This phenomenon is well known to take place in small metal aggregates, where the average bond length is shortened compared to the bulk because of the lower coordination number of the superficial atoms, which tend to compensate for the pending bonds by reducing their relative distance. Even Rh−Rh couples follow the same trend, especially if the average distances of the nanoalloys are compared with the ones of the pure Rh_19_ seeds. This however is not connected to a larger number of Rh constituting the nanocluster, since the seeds always have the same number of atoms; indeed this effect arises from the sole covering effect given by Au, which stimulates an elongation of the Rh−Rh bonds, allowing them to approach and reach the bulk PBE calculated value of 2.72 Å.^[56]^ To the best of our knowledge, this is the first time this stimulated bond elongation given by the shell enveloping is observed in AuRh nanosystems.

Finally, Au−Rh bonds are not sensibly affected by the size of the nanoalloys since their values remain always within the same error bars. The most remarkable differences involving DI and ID isomers are the ones relative to the magnetic moment per atom and to the energy gap between the frontier orbitals, namely the HOMO‐LUMO gap. Our simulations reveal that two isomers, which we may recall differ for the sole rotation of the Rh seed, can have different magnetic moments. According to our simulations, 39‐GS nanocluster in its ID form has an impressive magnetic moment per atom and remarkable large HOMO‐LUMO gap compared to its DI shaped counterpart. This suggests that small geometrical changes of the Rh seed could reflect into massive changes in the systems physical features and therefore, on its potential technological applications. Same considerations can be applied to the other analysed alloys and even on the Rh_19_ seed itself whose geometrical configurations strongly affect its electronic properties (see Table 1).

**Figure 6:**
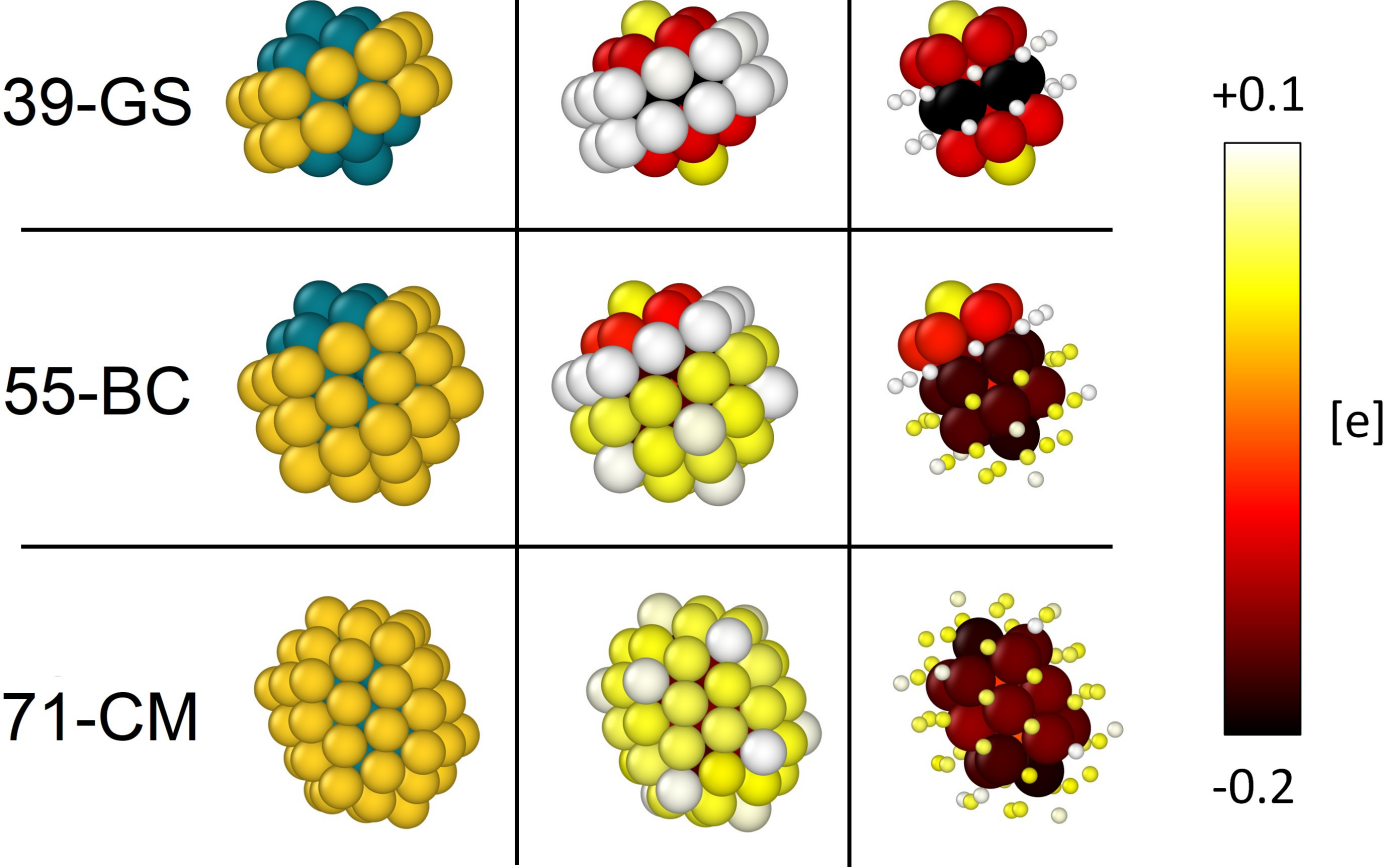
Left panel: morphologies from the classical MD and then DFT‐relaxed. Au is coloured in gold, Rh in blue. Central and right panels: distribution of charge imbalance, accordingly to the colour map. Right panel: Au atoms are shrunk to ease the visualisation of the Rh charge imbalances. Positive values in the colour map (white and yellow) indicate an excess of electrons, while negative values (orange, red and black) suggest charges loss. Au vertexes are positively charged, while Rh is more negative when covered by Au. Details on the calculations are given in the Computational methods section.

Since we are interested in characterising the optical and photophysical dynamics of these systems, we searched for the existence of charge imbalances within the structures which could allude to the existence of optically active charge transfer transitions. The analysis of the charge distribution within the most stable isomers is given in Figure 6.

The charge excesses and losses are given with respect to the number of electrons possessed by the single atomic species, as specified in the Computational methods section. By considering Figure 6, it can be clearly noticed the existence of a strong charge separation between Rh and Au atoms become self‐evident.

In particular, gold atoms naturally withdraw electrons from rhodium, resulting in a partially oxidised configuration where Rh atoms can lose up to 0.2 electrons.

Such an important charge imbalance directly arises from the electrochemical processes occurring at the Au−Rh interface which leads to a net electron transfer from rhodium to gold due to the difference in the electrochemical reduction potential.

The presence of partially oxidised Rh atoms in the external part of the clusters (in the case of 39‐GS and 55‐BC) could lead to interesting photocatalytic processes. Indeed, simple Coulombic interactions could favour the adsorption of electron rich molecules on top of these sites. Then by optically stimulating charge transfer transitions within the alloy, these adsorption points easily take part to the optical activity and this in turn would probably affect the vibrational and/or the electronic structure of the adsorbed molecule, activating chemical reactions. Such a mechanism could also involve the generation of hot‐carriers as already proposed for these nanoalloys.^[16]^

## Conclusions

In this work, we studied the formation and the physical properties of AuRh nanoalloys, between 20 and 150 atoms, by means of a multiscale protocol involving molecular MD and DFT simulations. Among the tested synthetic procedures, the one‐by‐one growth produces the most favourable geometries, especially when Au atoms are deposited over an Rh seed. Such processes generate structures with exotic shapes, whose growth is driven by the symmetry assumed by the Rh seed, and were found to have extremely interesting physical features. All simulations generate structures marked by a strong phase segregation presenting an Rh@Au core‐shell ordering. Any other arrangement was found to be highly unstable, sometimes even at room temperature (as in the case of randomly mixed chemical ordering). All tested AuRh nanoalloys are characterised by a remarkably high melting temperature, sometimes above 1000 K, even in cases where the Rh content is below 20 %. Moreover, we noted that structures presenting geometrical closures were, in general, more stable at higher temperatures. Ab‐initio investigations performed on structures built from an Rh_19_ seed revealed that the shape assumed by the inner parts of the nanostructures plays a major role in determining the system's physical features, especially regarding the magnetic moment and HOMO‐LUMO gap. An analysis of the charge distribution demonstrates the existence of a net charge separation within the nanoclusters, with gold atoms attracting electrons from rhodium. This suggests the existence of optically active charge transfer transitions that could potentially play a major role in the system's photocatalytic capabilities. To date these are nothing more than theoretical speculations, however we believe this route deserves to be explored since it could bring practical improvements in the use of AuRh nanosystems as effective photocatalysts. Globally, our results represent a solid step toward the comprehension of the dynamics and the physical properties of these notable nanoclusters.

## Computational Details

### Classical Molecular Dynamics Simulations

All calculations were performed as implemented in the open‐source package LoDiS^57^, using a potential derived in the second moment approximation to tight‐binging theory to describe the interatomic potential.[^58^, ^59^] This force field was proved to give reliable results for single and binary alloys at the nanoscale.[^40^, ^60^, ^61^] We consider the parametrization as in ref. [31]. By means of MD we studied three different synthetic mechanisms using the following methodologies:

1. One‐by‐one growth: we simulated the growth of Rh over a tetrahedron Au_20_ seed (Au_20_−Th) and of Au over various Rh seeds, namely double‐icosahedron Rh_19_ (Rh_19_−DI), truncated cuboctahedron Rh_38_ (Rh_38_−Co) and icosahedron Rh_55_ (Rh_55_−Ih). These structures are visible in the bottom panel of Figure 1. All simulation were conducted at 600 K, depositing one atom every 50 ns. The deposition stopped when the whole nanocluster reaches the sizes of 150 atoms. The choice of simulating Rh deposition on a gold seed in a single case comes from the evidence that Au naturally tends to diffuse towards the surface and constitute the external part of the particle, losing its role as kernel.
2. Nanocluster's coalescence: we simulated the collision and further coalescence of the gold‐based systems Au_20_−Th, Au_38_‐Co, Au_55_−Ih and Au_75_−Co with the rhodium Rh_19_−DI, Rh_55_−Ih and Rh_75_−Co nanoclusters. This allowed us to obtain and analyze the dynamics of formation of several alloyed structures that differ in sizes and compositions. The collision always occurs along the same cartesian axis, thus using the same nanoclusters impact angle. All systems were simulated at 600 K for a total amount of 500 ns, allowing the system to relax after the impact which always takes place within the first 50 ns. Snapshots showing coalesced structures are reported within the graphs in Figure 2.
3. Nanodroplets annealing: we simulated the annealing process of several Au, Rh and AuRh systems. In particular we tested the following structures: Au_55_−Ih, Au_147_−Ih; Rh_55_−Ih, Rh_147_−Ih; Rh@Au core‐shell 55 atoms Au_42_Rh_13_−Ih and Au_36_Rh_19_−Ih, 71 atoms Au_52_Rh_19_, 92 atoms Au_73_Rh_19_, 147 atoms Au_92_Rh_55_−Ih, Au_128_Rh_19_−Ih and Au_134_Rh_13_−Ih; Au@Rh inverse core‐shell 55 atoms Au_13_Rh_42_−Ih; randomly mixed chemical ordered 55 atoms Au_26_Rh_29_−Ih; Janus ordered 55 atoms Au_27_Rh_28_−Ih. Notice that most of the chosen nanoalloys come from the simulations described in 1) and 2) while other structures were built *ad hoc* to be tested in these simulations and to determine their T_m_. The systems were first melted by gradually increasing the temperature and then cooled down using an heating (and cooling) rate of 1 K/ns, which is small enough to consider the solid‐liquid transitions as a reversible process and was proven to give reliable T_m_ within a ±50 K range.^[62]^ The initial and final temperatures depend on each system, and are chosen in order to make the solid‐liquid transition clearly visible from the excess energy data, as shown in Figure 3. Please notice that some of the mentioned nanoclusters are not explicitly treated in the discussion and not considered in the plot reported in Figure 4. This is because, as mentioned in the text, some of the considered 55 atoms structures (inverse core‐shell Au_13_Rh_42_, randomly mixed chemical ordered Au_26_Rh_29_−Ih and Janus ordered Au_27_Rh_28_−Ih) are highly unstable and naturally assume core‐shell motifs during the initial heating, giving caloric patterns and structure similar to the core‐shell cases (e. g. Au_42_Rh_13_−Ih or Au_36_Rh_19_−Ih). The caloric profiles of these particular alloys are attached as Supporting Information.

Before performing the simulations, all nanoclusters were quenched through a 5 ns long equilibration. The simulations were performed via the Velocity‐Verlet, using a time‐step of 5 fs and imposing a temperature control through an Andersen thermostat with a stochastic collision frequency of 5 ⋅ 10^11^ Hz. This protocol has already been assessed as reliable by other simulations involving metallic nanoclusters.[^60^, ^63^, ^64^] All described simulations were repeated 10 times, randomly assigning the initial value of the atomic velocities from a fixed‐temperature Maxwell‐Boltzmann distribution. All results we reported in this work are averaged over the 10 trajectories, and thus minimise spurious contributions coming from the initial conditions of the systems. Lastly, most of our calculations are performed on geometrically closed shapes since they are the most energetically favourable in the case of bare metallic nanoclusters.^[46]^

### Density Functional Theory calculations

As discussed in the Result and Discussion section, we performed ground‐state DFT calculations on the most favorable geometries recovered along the MD simulations and relative isomers. These calculations were conducted at the PBE level^55^ using plane‐waves basis sets, as implemented in the code Quantum ESPRESSO version 6.6.[^65^, ^66^] To describe the core electrons of the metal species we employed fully relativistic norm‐conserving pseudopotentials. For both Au and Rh, the kinetic energy cut‐off for the wavefunction and the electronic density were converged to 60 Ry and 300 Ry respectively. The various structures were relaxed through spin‐polarized BFGS optimizations, assigning a non‐null magnetization to both Au and Rh species of 0.5 bohr/atoms and imposing a 0.01 Ry gaussian smearing to facilitate the convergence of the electronic densities. Calculations were performed at Γ point using a system‐dependent cubic cell large enough to assure at least a 15 Å vacuum layer among a nanocluster and its replica. A subset of the structures relaxed with this approach are visible in Figure 5, while the geometrical coordinates of all optimized nanoclusters are attached as Supporting Information.

The effective atomic charges assigned on each atom of the nanoalloys were calculated as $Q_{eff}=n_{e}-q\;$ where $n_{e}$ is the number of accounted valence electrons for Rh and Au, while q is the Bader charge calculated through the Bader partitioning charge analysis.[^67^, ^68^, ^69^] Bader charges estimation were performed using a code specifically developed by the Henkelman Research Group, University of Texas at Austin (Texas, USA).^[70]^

### Energetics and Characterisation

To characterise the thermodynamics of the trajectories and estimate the stabilities of various isomers, we exploited some useful quantities that allow a characterization of the processes and systems energetics. These quantities are defined as follows:

• Excess energy: this represents the energy required to form a certain alloyed structure starting from the Au and Rh bulk phases. This quantity was proved to be a reliable descriptor for the relative stability of alloys with different sizes and compositions.[^32^, ^46^, ^71^] It is defined as:

(1) $$E_{exc}=\frac{\left[N_{Au}E_{Au}^{coh}+N_{Rh}E_{Rh}^{coh}\right]-E_{alloy}}{{\left(N_{Au}+N_{Rh}\right)}^{2/3}}$$

where $N_{Au}$ and $N_{Rh}$ are the number of Au and Rh atoms constituting the alloy, $E_{Au}^{coh}$ and $E_{Rh}^{coh}$ are the cohesive energy of bulk Au and Rh and $E_{alloy}$ is the total energy of the nanoalloy, estimated classically by MD. In all cases we impose $E_{Au}^{coh}$ =3.55 eV and $E_{Rh}^{coh}$ =5.75 eV which are the values of cohesive energies predicted by the adopted force field.

• Binding energy: the energy gained by the systems when the atoms bind together starting from their gas phase, defined as:

(2) $$E_{bond}=\frac{\left[N_{Au}E_{Au}^{free}+N_{Rh}E_{Rh}^{free}\right]-E_{alloy}}{N_{Au}+N_{Rh}}$$

where $E_{Au}^{free}$ and $E_{Rh}^{free}$ are the energy of single Au and Rh atoms in the gas phases, estimated at the DFT level. In this case $E_{alloy}$ refers to the total energy of the relaxed nanoalloy, calculated at the DFT level.

• Mixing energy: energy gained by the systems when alloys with specific geometries are formed, compared to the pure metal nanoclusters. It is defined as:

(3) $$\;\epsilon_{mix}=\frac{\left[\frac{N_{Au}}{N_{Au}+N_{Rh}}E_{Au}^{alloy}+\frac{N_{Rh}}{N_{Au}+N_{Rh}}E_{Rh}^{alloy}\right]-E_{alloy}}{N_{Au}+N_{Rh}}$$

where $E_{Au}^{alloy}$ and $E_{Rh}^{alloy}$ are the DFT calculated energy of pure Au and Rh nanoclusters at the geometry of the considered alloy. Even in this case $E_{alloy}$ is the total energy of the relaxed nanoalloy computed at the DFT level.

The $\epsilon_{mix}\;$ values collected in Table 1, are the mixing energy defined in Equation (3) normalized by the number of atoms.

Finally, the graphical representations of all nanocluster structures were obtained using the visualization code OVITO.^[72]^

## Conflict of interest

The authors declare no conflict of interest.

1

## Supporting information

As a service to our authors and readers, this journal provides supporting information supplied by the authors. Such materials are peer reviewed and may be re‐organized for online delivery, but are not copy‐edited or typeset. Technical support issues arising from supporting information (other than missing files) should be addressed to the authors.

Supporting Information

## Data Availability

The data that support the findings of this study are available from the corresponding author upon reasonable request.

## References

[1] I. Chorkendorff, J. W. Niemantsverdriet, *Concepts of Modern Catalysis and Kinetics*, Wiley-VCH, Weinheim, Germany, 3rd Edition, **2003**.

[2] R. Jin , Nanoscale 2015, 7, 1549–1565. 25532730 10.1039/c4nr05794e

[3] I. Chakraborty , T. Pradeep , Chem. Rev. 2017, 117, 8208–8271. 28586213 10.1021/acs.chemrev.6b00769

[4] M. Vanzan , S. Corni , J. Phys. Chem. A 2018, 122, 6864–6872. 30074789 10.1021/acs.jpca.8b01797

[5] O. J. H. Chai , Z. Liu , T. Chen , J. Xie , Nanoscale 2019, 11, 20437–20448. 31657426 10.1039/c9nr07272a

[6] M. Vanzan , T. Cesca , B. Kalinic , C. Maurizio , G. Mattei , S. Corni , ACS Photonics 2021, 8, 1364–1376.

[7] A. S. Lapp , Z. Duan , N. Marcella , L. Luo , A. Genc , J. Ringnalda , A. I. Frenkel , G. Henkelman , R. M. Crooks , J. Am. Chem. Soc. 2018, 140, 6249–6259. 29750512 10.1021/jacs.7b12306

[8] G. W. Piburn , H. Li , P. Kunal , G. Henkelman , S. M. Humphrey , ChemCatChem 2018, 10, 329–333.

[9] V. Amendola , V. Torresan , D. Forrer , A. Guadagnini , D. Badocco , P. Pastore , M. Casarin , A. Selloni , D. Coral , M. Ceolin , M. B. Fernandez Van Raap , A. Busato , P. Marzola , A. E. Spinelli , ACS Nano 2020, 14, 12840–12853. 32877170 10.1021/acsnano.0c03614 PMC8011985

[10] D. T. L. Alexander , D. Forrer , E. Rossi , E. Lidorikis , S. Agnoli , G. D. Bernasconi , J. Butet , O. J. F. Martin , V. Amendola , Nano Lett. 2019, 19, 5754–5761. 31348861 10.1021/acs.nanolett.9b02396

[11] S. Seraj , P. Kunal , H. Li , G. Henkelman , S. M. Humphrey , C. J. Werth , ACS Catal. 2017, 7, 3268–3276.

[12] L. Luo , Z. Duan , H. Li , J. Kim , G. Henkelman , R. M. Crooks , J. Am. Chem. Soc. 2017, 139, 5538–5546. 28387511 10.1021/jacs.7b01653

[13] H. Okamoto , T. B. Massalski , Bull. Alloy Phase Diagrams 1984, 5, 384–387.

[14] P. Villars, Au−Rh Binary Phase Diagram 0–100 at. % Rh, https://materials.springer.com/isp/phase-diagram/docs/c_0900246.

[15] M. Jansen , Angew. Chem. Int. Ed. 1987, 26, 1098–1110;

[16] N. Kang , Q. Wang , R. Djeda , W. Wang , F. Fu , M. M. Moro , M. D. L. A. Ramirez , S. Moya , E. Coy , L. Salmon , J. L. Pozzo , D. Astruc , ACS Appl. Mater. Interfaces 2020, 12, 53816–53826. 10.1021/acsami.0c16247 33201661

[17] H. Li , L. Luo , P. Kunal , C. S. Bonifacio , Z. Duan , J. C. Yang , S. M. Humphrey , R. M. Crooks , G. Henkelman , J. Phys. Chem. C 2018, 122, 2712–2716.

[18] D. Kim , H. Nam , Y. H. Cho , B. C. Yeo , S. H. Cho , J. P. Ahn , K. Y. Lee , S. Y. Lee , S. S. Han , ACS Catal. 2019, 9, 8702–8711.

[19] X. Wang , H. Wang , N. Maeda , A. Baiker , Catalysts 2019, 9, 937.

[20] X. Wang , N. Maeda , D. M. Meier , A. Baiker , ChemCatChem 2021, 13, 438–444.

[21] Z. Konuspayeva , P. Afanasiev , T. S. Nguyen , L. Di Felice , F. Morfin , N. T. Nguyen , J. Nelayah , C. Ricolleau , Z. Y. Li , J. Yuan , G. Berhault , L. Piccolo , Phys. Chem. Chem. Phys. 2015, 17, 28112–28120. 25765742 10.1039/c5cp00249d

[22] S. García , L. Zhang , G. W. Piburn , G. Henkelman , S. M. Humphrey , ACS Nano 2014, 8, 11512–11521. 25347078 10.1021/nn504746u

[23] R. Camposeco , M. Hinojosa-Reyes , S. Castillo , N. Nava , R. Zanella , Environ. Sci. Pollut. Res. Int. 2020, 28, 10734–10748. 33099755 10.1007/s11356-020-11341-7

[24] X. Zhao , Y. Jia , R. Dong , J. Deng , H. Tang , F. Hu , S. Liu , X. Jiang , Chem. Commun. 2020, 56, 10918–10921. 10.1039/d0cc03481a 32808607

[25] Z. Konuspayeva , G. Berhault , P. Afanasiev , T. S. Nguyen , S. Giorgio , L. Piccolo , J. Mater. Chem. A 2017, 5, 17360–17367.

[26] L. Piccolo , Z. Y. Li , I. Demiroglu , F. Moyon , Z. Konuspayeva , G. Berhault , P. Afanasiev , W. Lefebvre , J. Yuan , R. L. Johnston , Sci. Rep. 2016, 6, 35226. 27739480 10.1038/srep35226 PMC5064371

[27] L. Vitos , A. V. Ruban , H. L. Skriver , J. Kollár , Surf. Sci. 1998, 411, 186–202.

[28] G. Wang , Y. Xu , P. Qian , Y. Su , Comput. Mater. Sci. 2021, 186, 110002.

[29] I. Demiroglu , Z. Y. Li , L. Piccolo , R. L. Johnston , Catal. Sci. Technol. 2016, 6, 6916–6931.

[30] I. Demiroglu , T. E. Fan , Z. Y. Li , J. Yuan , T. D. Liu , L. Piccolo , R. L. Johnston , Faraday Discuss. 2018, 208, 53–66. 29796531 10.1039/c7fd00213k

[31] G. Rossi , R. Ferrando , Comput. Theor. Chem. 2017, 1107, 66–73.

[32] F. Baletto , R. Ferrando , Rev. Mod. Phys. 2005, 77, 371–423.

[33] C. Castillo-Quevedo , C. E. Buelna-Garcia , E. Paredes-Sotelo , E. Robles-Chaparro , E. Zamora-Gonzalez , M. F. Martin-Del-campo-solis , J. M. Quiroz-Castillo , T. Del-Castillo-Castro , G. Martínez-Guajardo , A. De-Leon-flores , M. Cortez-Valadez , F. Ortiz-Chi , T. Gaxiola , S. J. Castillo , A. Vásquez-Espinal , S. Pan , J. L. Cabellos , Molecules 2021, 26, 5710.34577181

[34] C. E. Buelna-García , E. Robles-Chaparro , T. Parra-Arellano , J. M. Quiroz-Castillo , T. Del-Castillo-Castro , G. Martínez-Guajardo , C. Castillo-Quevedo , A. De-León-flores , G. Anzueto-Sánchez , M. F. Martin-Del-campo-solis , A. M. Mendoza-Wilson , A. Vásquez-Espinal , J. L. Cabellos , Molecules 2021, 26, 3953. 34203563 10.3390/molecules26133953 PMC8271876

[35] Z. Valizadeh , M. Abbaspour , J. Phys. Chem. Solids 2020, 144, 109480.

[36] P. C. Chen , M. Gao , S. Yu , J. Jin , C. Song , M. Salmeron , M. C. Scott , P. Yang , Nano Lett. 2021, 21, 6684–6689. 34283612 10.1021/acs.nanolett.1c02225

[37] Y. Shubin , P. Plyusnin , M. Sharafutdinov , E. Makotchenko , S. Korenev , Nanotechnology 2017, 28, 205302. 28383287 10.1088/1361-6528/aa6bc9

[38] A. S. Chaves , M. J. Piotrowski , J. L. F. Da Silva , Phys. Chem. Chem. Phys. 2017, 19, 15484–15502. 28580970 10.1039/c7cp02240a

[39] F. Buendía , J. A. Vargas , R. L. Johnston , M. R. Beltrán , Comput. Theor. Chem. 2017, 1119, 51–58.

[40] R. Ferrando , J. Jellinek , R. L. Johnston , Chem. Rev. 2008, 108, 845–910. 18335972 10.1021/cr040090g

[41] F. Calvo, *Nanoalloys: From Fundamentals to Emergent Applications*, Elsevier, **2013**.

[42] J. Li , X. Li , H. J. Zhai , L. S. Wang , Science. 2003, 299, 864–867. 12574622 10.1126/science.1079879

[43] C. C. Chang , J. J. Ho , Phys. Chem. Chem. Phys. 2014, 16, 5393–5398. 24499741 10.1039/c3cp54667e

[44] W. R. Tyson , Can. Metall. Q. 1975, 14, 307–314.

[45] O. López-Estrada , E. Orgaz , F. Baletto , J. Mater. Chem. C 2020, 8, 2533–2541.

[46] F. Baletto , J. Phys. Condens. Matter 2019, 31, 113001. 30562724 10.1088/1361-648X/aaf989

[47] W. J. Debonte , J. M. Poate , Thin Solid Films 1975, 25, 441–448.

[48] K. Palotás , L. Óvári , G. Vári , R. Gubó , A. P. Farkas , J. Kiss , A. Berkó , Z. Kónya , J. Phys. Chem. C 2018, 122, 22435–22447. 10.1039/c8cp00790j 29799587

[49] T. P. Rossi , P. Erhart , M. Kuisma , ACS Nano 2020, 14, 9963–9971. 32687311 10.1021/acsnano.0c03004 PMC7458472

[50] D. Nelli , C. Roncaglia , R. Ferrando , C. Minnai , J. Phys. Chem. Lett. 2021, 12, 4609–4615. 33971714 10.1021/acs.jpclett.1c00787

[51] D. Nelli , M. Cerbelaud , R. Ferrando , C. Minnai , Nanoscale Adv. 2021, 3, 836–846. 10.1039/d0na00891e PMC9416879 36133833

[52] P. Sahu , B. L. V. Prasad , Langmuir 2014, 30, 10143–10150. 25111614 10.1021/la500914j

[53] D. R. Lide, W. M. M. Haynes, G. Baysinger, L. I. Berger, H. V. Kehiaian, D. L. Roth, D. Zwillinger, M. Frenkel, R. N. Goldberg, *CRC Handbook of Chemistry and Physics*, **2010**.

[54] R. Pinal , Org. Biomol. Chem. 2004, 2, 2692–2699. 15351835 10.1039/B407105K

[55] J. P. Perdew , K. Burke , M. Ernzerhof , Phys. Rev. Lett. 1996, 77, 3865–3868. 10062328 10.1103/PhysRevLett.77.3865

[56] P. Janthon , S. Luo , S. M. Kozlov , F. Viñes , J. Limtrakul , D. G. Truhlar , F. Illas , J. Chem. Theory Comput. 2014, 10, 3832–3839. 26588528 10.1021/ct500532v

[57] *LoDiS software*, https://github.com/kcl-tscm/LoDiS.

[58] V. Rosato , M. Guillope , B. Legrand , Philos. Mag. A 1989, 59, 321–336.

[59] F. Cleri , V. Rosato , Phys. Rev. B 1993, 48, 22–33. 10.1103/physrevb.48.22 10006745

[60] L. Delgado-Callico , K. Rossi , R. Pinto-Miles , P. Salzbrenner , F. Baletto , Nanoscale 2021, 13, 1172–1180. 33404027 10.1039/d0nr06850k

[61] F. Baletto , C. Mottet , R. Ferrando , Chem. Phys. Lett. 2002, 354, 82–87.

[62] K. Rossi , L. Bartok-Pártay , G. Csányi , F. Baletto , Sci. Rep. 2018, 8, 1–9. 30087376 10.1038/s41598-018-29408-4 PMC6081399

[63] D. Schebarchov , F. Baletto , D. J. Wales , Nanoscale 2018, 10, 2004–2016. 29319705 10.1039/c7nr07123j PMC5901115

[64] M. Vanzan , M. Rosa , S. Corni , Nanoscale Adv. 2020, 20, 2842–2852. 10.1039/d0na00213e PMC9417423 36132411

[65] P. Giannozzi , S. Baroni , N. Bonini , M. Calandra , R. Car , C. Cavazzoni , D. Ceresoli , G. L. Chiarotti , M. Cococcioni , I. Dabo , A. D. Corso , G. Fratesi , S. De Gironcoli , R. Gebauer , U. Gerstmann , C. Gougoussis , A. Kokalj , L. Martin-Samos , N. Marzari , F. Mauri , R. Mazzarello , S. Paolini , A. Pasquarello , L. Paulatto , C. Sbraccia , S. Scandolo , A. P. Seitsonen , A. Smogunov , P. Umari , R. M. Wentzcovitch , J. Phys. Condens. Matter 2009, 21, 395502. 21832390 10.1088/0953-8984/21/39/395502

[66] P. Giannozzi , O. Andreussi , T. Brumme , O. Bunau , M. Buongiorno , M. Marsil , L. Paulatto , D. Rocca , J. Phys. Condens. Matter 2017, 29, 465901. 29064822 10.1088/1361-648X/aa8f79

[67] G. Henkelman , A. Arnaldsson , H. Jónsson , Comput. Mater. Sci. 2006, 36, 354–360.

[68] E. Sanville , S. D. K. Kenny , R. Smith , G. Henkelman , J. Comput. Chem. 2007, 28, 899–908. 17238168 10.1002/jcc.20575

[69] W. Tang , E. Sanville , G. Henkelman , J. Phys. Condens. Matter 2009, 21, 84204. 10.1088/0953-8984/21/8/084204 21817356

[70] *Bader Charge Analysis*, http://theory.cm.utexas.edu/henkelman/code/bader/.

[71] M. Settem , J. Alloys Compd. 2020, 844, 155816.

[72] A. Stukowski , Model. Simul. 2010, 18, 015012.

