# Supplementary material for: Exploring AuRh Nanoalloys: A Computational Perspective on the Formation and Physical Properties
Source: Chemphyschem. 2022 Mar 14;23(8):e202200035. doi: 10.1002/cphc.202200035 (PMC9314847; doi:10.1002/cphc.202200035)
Supplement: Supplementary file 1 — Supporting Information [file CPHC-23-0-s001.pdf]

ChemPhysChem

Supporting Information

Exploring AuRh Nanoalloys: A Computational Perspective on the Formation and Physical Properties

Mirko Vanzan, Robert M. Jones, Stefano Corni, Roberto D'Agosta, and Francesca Baletto*

Contents

-Figure S1. Caloric plot for 55 atoms inverse core-shell ordering alloy

-Figure S2. Caloric plot for 55 atoms janus ordered alloy

-Figure S3. Caloric plot for 55 atoms randomly mixed ordered alloy

-Geometrical coordinates of DFT-PBE optimized notable nanoclusters

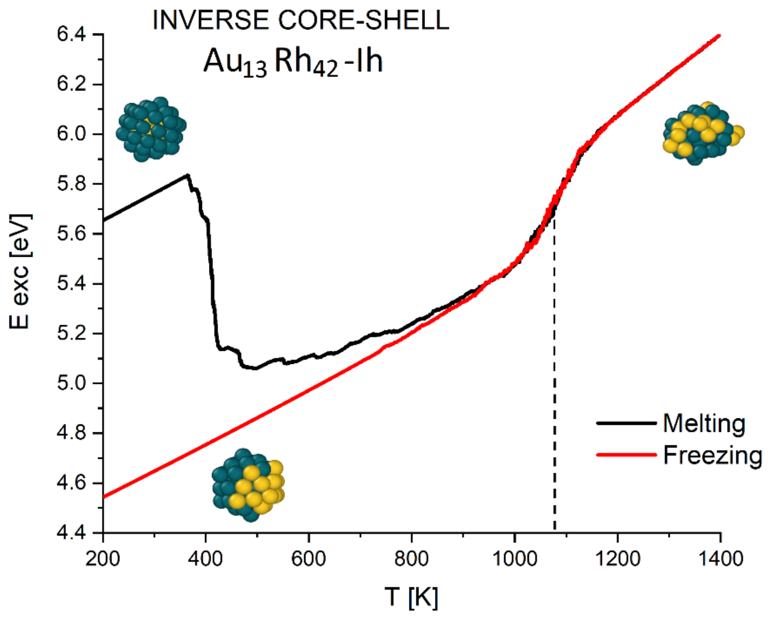

Figure S1. Excess energy as function of temperature for the annealing of the inverse core-shell icosahedral $Au_{13}Rh_{42}$ nanocluster.

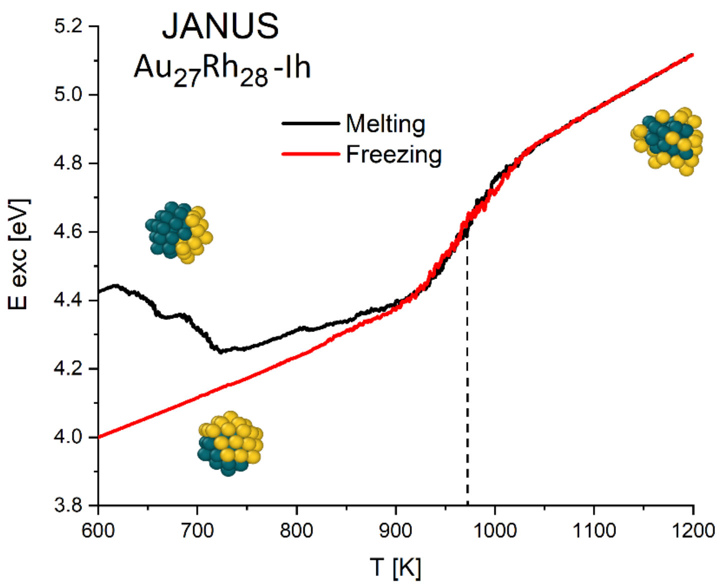

Figure S2. Excess energy as function of temperature for the annealing of the janus ordered icosahedral $Au_{27}Rh_{28}$ nanocluster.

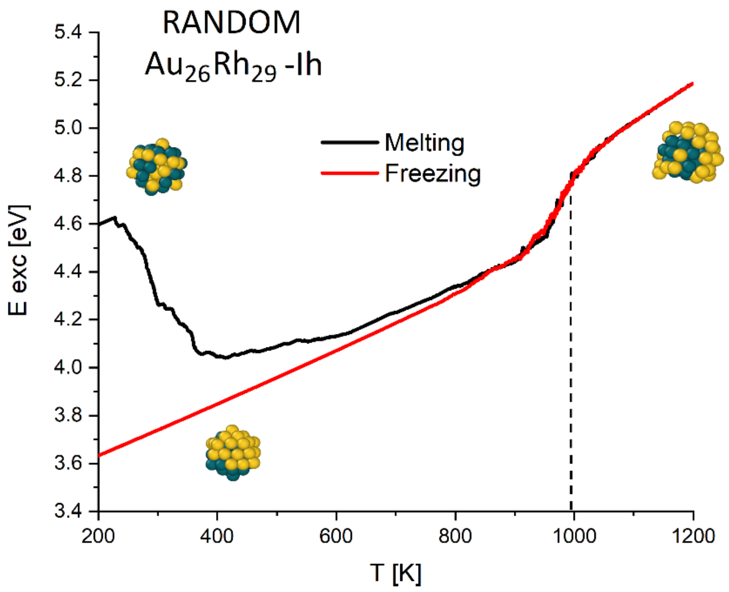

Figure S3. Excess energy as function of temperature for the annealing of the randomly mixed icosahedral $Au_{26}Rh_{29}$ nanocluster.

39-GS_ID

Rh	-0.0352979765	3.0140688649	-1.5500018561
Rh	-1.5614733710	0.3607368204	1.6236749197
Rh	-2.3442702167	1.9387918065	-2.4580732034
Rh	-1.5002323724	-1.7138834324	-0.0721556153
Rh	-0.8189751853	0.6776895300	-0.8569187042
Rh	0.7933046036	-0.6572269741	0.8332676202
Rh	-2.0083791229	-0.7425186321	-2.6250624346
Rh	2.0534221208	0.7009099843	2.6721790648
Rh	1.7633798038	0.9463280371	-1.0908494495
Rh	0.0732308950	-3.0453786412	1.5991028697
Rh	0.4595000659	1.9952519379	1.0000367443
Rh	0.0325842754	0.9175168696	-3.2604813104
Rh	-3.3334032842	0.3284176811	-0.5225021206
Rh	3.3730604280	-0.3649258543	0.5687847838
Rh	0.5589301924	-1.3560755561	-1.7443384616
Rh	2.1542553104	-2.6816711841	-0.0927336693
Rh	2.3101992676	-1.9145969929	2.4275704829
Rh	0.0102208030	-0.9562276884	3.3060128717
Rh	-2.1119081776	2.6487770970	0.1422324563
Au	-0.6631970799	-2.9039295538	-3.6672399636
Au	3.3355625530	2.4399379955	0.8529164911
Au	-2.3900253170	0.0305703858	4.2840519460
Au	2.0379909651	-3.5414310681	-2.7199859403
Au	1.4804539589	-1.1652446679	-4.3295879083
Au	3.4384875976	-1.1978739099	-2.1248819535
Au	-0.2843663484	1.8034689582	3.8051019767
Au	-2.8076843850	-3.2797540931	-1.9295207401
Au	4.5303831541	1.2585727545	-1.3697714138
Au	1.8324854491	3.4399189651	2.9876758220
Au	-0.1116703470	-4.0876593266	-1.0141962889
Au	-4.2587619750	0.0060959275	2.0439637849
Au	-4.1969285121	-2.1528400050	0.2790694195
Au	-0.8660627140	4.1405343403	2.0909825687
Au	2.7326075295	1.2311655318	-3.6653658118
Au	-2.9790373627	2.4453269725	2.7428663815
Au	-2.2630092361	-4.2814964403	0.7636162033
Au	1.3285706421	4.5089191737	0.3140201978
Au	2.6582188529	3.4344670697	-1.8843454532
Au	-2.4220454839	-2.2259826827	2.6423356933

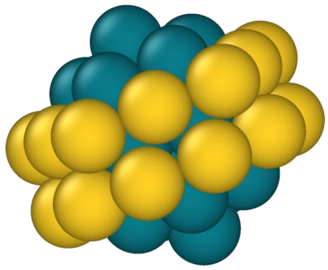

39-GS_DI

Rh	-1.5060416601	-2.1600716891	2.1787961298
Rh	-1.5715990778	0.1605593877	-1.6795798133
Rh	-2.8555041161	-0.0301422584	2.6852258924
Rh	-0.4402714579	2.2475489252	-0.3878331464
Rh	-0.8994170234	-0.0112622872	0.8732200641
Rh	0.8479727389	0.0105691179	-0.7920058294
Rh	-1.3330807792	2.0232845120	2.4106750310
Rh	3.3712821304	-0.1487036114	-0.0911716279
Rh	1.2514156449	-1.4865510880	1.3012080976
Rh	0.5338189645	-1.1657890707	-3.0950025612
Rh	-0.6154038590	-2.1437852223	-0.6370571418
Rh	-0.3612238822	-0.1680913107	3.3905676935
Rh	-3.0601467055	1.3805058348	0.5364647181
Rh	2.4408528405	2.0248588929	-1.1101087305

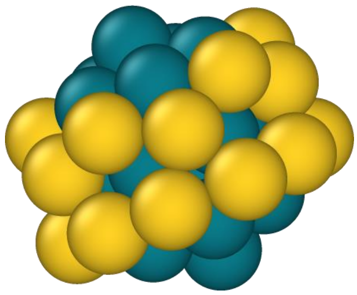

Rh	1.3587152759	1.2390819176	1.4383799676
Rh	0.6513156038	1.4460077634	-2.9502290161
Rh	2.7497144905	0.0259322170	-2.6047169245
Rh	2.2517306122	-2.0934818636	-1.3510177821
Rh	-3.1599471232	-1.1882802513	0.3966007100
Au	-3.1605101384	-2.3412726802	-2.0105968619
Au	1.2677527534	4.3294455166	-0.8842048981
Au	2.2780840541	-0.3015883051	3.7440481003
Au	-0.8807661385	-3.4385699662	-3.1925894018
Au	-1.8425434217	1.6533806446	-4.0245314771
Au	-4.2211307320	0.2636780604	-1.7468777211
Au	3.9636733674	-1.6903595607	2.0331114100
Au	0.1273858109	-4.0742427464	1.3204616704
Au	-2.9561519780	2.7882424299	-1.7309456160
Au	-2.2291826874	3.9727706039	0.7154231065
Au	-2.5233887018	-3.8527906466	0.2703294224
Au	0.7566959126	-2.6832322847	3.6907074736
Au	2.8338044876	-3.7305512207	0.6687007110
Au	3.1051589340	3.4274180862	1.0790344303
Au	-1.9613815285	-1.0454002310	-4.1592708782
Au	0.9844422635	2.2060005208	3.9276905403
Au	0.8999323196	-4.3103526145	-1.3712125902
Au	0.4269287155	3.9184598728	1.7690267750
Au	-0.5754471828	3.8047568800	-2.7862008809
Au	4.0524412731	1.1420277253	2.1754509546

55-BC_ID

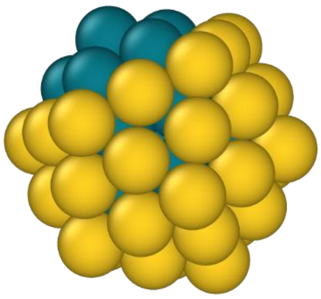

Rh	0.2023948628	-2.0880904413	-1.5257272579
Rh	-2.3821080046	0.5914837761	0.8557773104
Rh	-1.1335647393	2.1300313571	-1.0395795299
Rh	-1.9654791434	-0.4165228745	-1.6472813749
Rh	1.0884336055	-2.0622876839	1.0107709970
Rh	1.5677747910	2.0024543029	-0.5302614843
Rh	2.3712913658	-0.6020457876	-0.8417273249
Rh	0.4749189399	0.4647303832	-2.4965336212
Rh	2.1634881514	-4.1004743799	2.0030407860
Rh	0.6559500619	-2.6157825342	3.5450683441
Rh	-0.0201884478	0.0316474586	-0.0106904563
Rh	3.5012510316	-2.7415576544	0.2067553042
Rh	3.0813583077	-1.7473893885	2.7000837929
Rh	1.3361505423	-4.2227549254	-0.4813007109
Rh	-1.5585557911	-2.0098308226	0.5432298741
Rh	-0.2038164464	2.0858458383	1.5433511614
Rh	1.9545014391	0.4015570599	1.6573543628
Rh	-0.4234890500	-4.1457375485	1.5834356244
Rh	-0.4799510709	-0.4703751130	2.5011699185
Au	-0.7237108355	1.7072310065	4.3103814933
Au	0.9968705919	0.9353833013	-5.1047286875
Au	-3.7389741382	-2.6027872844	-1.1613492068
Au	-1.3947538239	4.4641051197	0.5470675510
Au	0.7318078981	-1.7486065099	-4.2820525925
Au	-1.4384805747	-4.3971120298	-1.0526819784
Au	-2.1730963628	-2.6849131373	3.2744883559
Au	-4.1758124641	-1.5322886750	1.5031019744
Au	-4.0309934194	-0.8862240925	-3.3817156949
Au	4.6370393033	-0.2265237120	0.8802890559

Au	3.2463565726	4.0990822817	-1.0859670856
Au	-0.9579084532	-1.0206871495	5.1306806864
Au	4.8906902416	-1.2916262534	-1.7051962398
Au	-3.1692058332	-4.1843087198	1.1414851346
Au	3.7554710889	2.5456202462	1.2152063563
Au	-4.8753158039	1.1955113286	1.7713903823
Au	-1.5811619683	0.0233964569	-4.4252523711
Au	1.5906469503	-0.0903452619	4.4546263624
Au	-3.2966778035	1.7836934546	-2.8460575685
Au	-1.8556437584	-2.6796396594	-3.3831922107
Au	4.2092194006	1.4716129645	-1.4537519843
Au	0.4937855217	4.3640929667	-1.6771938255
Au	-2.3056309241	4.3390788921	-2.1244593600
Au	-3.0348404569	0.1271572378	3.5949469514
Au	-3.7336065631	2.8676458879	-0.1809737121
Au	-4.6285718547	0.1426900749	-0.8273499242
Au	1.8807372066	2.6330903667	3.4083481060
Au	-0.4052823069	4.2702312842	3.1811529693
Au	2.2269470639	2.6114716066	-3.2355248959
Au	3.0608240545	-0.1642802318	-3.5563706640
Au	-0.6861523006	2.7316531619	-3.7738883255
Au	1.4754163035	4.3467423416	1.0858956327
Au	4.0449106012	0.7611343092	3.4312568659
Au	0.4509915617	-4.3401881115	-3.1161490921
Au	-2.7551211874	2.8517364901	2.5717400469
Au	2.7769760675	-2.9010109735	-2.536578220

55-BC_DI

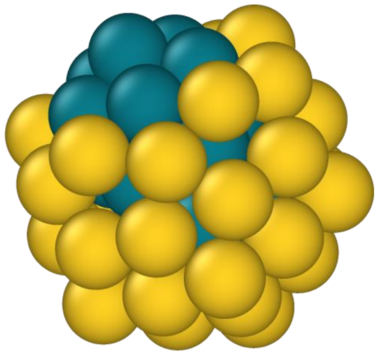

Rh	2.3533479829	1.0295052900	0.1424637531
Rh	-1.5781074514	0.8673119188	-1.8569827759
Rh	-2.0258911913	1.4615792583	0.7831368154
Rh	0.1491816293	2.4881332087	-0.5280506202
Rh	1.8834705921	-1.4147296203	-0.7361428917
Rh	-1.1289996942	-0.6782805430	2.2379408537
Rh	1.6244849967	-0.9520590181	1.8740335018
Rh	0.4243535973	1.5378636399	2.0132062953
Rh	3.9445427195	-2.7773589828	-1.5098891156
Rh	1.5649590757	-3.7932511635	-1.6083437373
Rh	-0.0155788241	-0.0365259548	-0.0039700576
Rh	4.1598553799	-1.1508913676	0.4794136169
Rh	2.7395665120	-3.3609281544	0.7015109271
Rh	3.8615158882	-0.2285828300	-1.9394205111
Rh	1.1455525503	0.6182497458	-2.2774327952
Rh	-2.3630740739	-1.0937428403	-0.1519954775
Rh	-0.1358959124	-2.5984335802	0.5236129545
Rh	2.2762065947	-1.8566581145	-3.2481310530
Rh	-0.4111798359	-1.6518178539	-2.0689507753
Au	-3.0371042319	-2.7521009715	-2.3129859699
Au	0.8401334064	3.2477434949	4.1458095180
Au	1.2728497988	3.3544504580	-3.0011517351
Au	-4.6612058265	0.4408839094	0.6868615038
Au	2.9253856144	2.8693376900	2.2792701148
Au	3.7614030154	2.3625858537	-2.4299143994
Au	0.3883078868	-0.8248899674	-4.7951267373
Au	-0.5328516879	1.7078240119	-4.3314681256
Au	0.3258957704	5.1384156251	-1.0620453754

Au	1.3174319782	-3.6883193352	2.9279628085
Au	-2.3755423959	-1.3264001599	4.6194087626
Au	0.1768856378	-3.5180390968	-3.9861301722
Au	3.7397196385	-2.2206843988	3.0289822466
Au	2.8002302149	0.6251563204	-4.4114598291
Au	-1.4298776855	-3.3883618827	2.9212119617
Au	-3.2936543691	1.8838291445	-3.7853330389
Au	0.6008197755	4.3674171197	1.5968520296
Au	-1.0101043654	-4.4771742745	-1.5994553248
Au	-1.9773814666	4.2628715631	0.2886617528
Au	2.6956636511	3.7917408080	-0.3433457247
Au	0.4244494588	-1.5637331564	4.4071946545
Au	-3.3693599019	0.8794525485	3.2235336042
Au	-4.1393942894	3.0509606574	1.6158009763
Au	-2.2581186119	-0.7288110651	-4.0644864662
Au	-3.8305526638	2.5462913513	-1.1249492057
Au	-1.5568723525	3.6723111479	-2.5147233150
Au	-2.7080670459	-3.8107423222	0.4648383906
Au	-4.8944047715	-2.1834598765	-0.2795618109
Au	-0.8059175900	1.0097021384	4.5501350901
Au	2.0392461968	0.6923399954	4.1647640137
Au	-1.7010907980	3.2461699069	2.9816907356
Au	-3.7668607196	-1.8233562451	2.2541213660
Au	0.6333253464	-5.1566328877	0.6215662686
Au	4.9526536438	1.5519746341	-0.0012972592
Au	-4.2606494479	-0.1442619005	-2.0993793623
Au	4.2462686515	0.4961261237	2.5381591463

$Au_{42}Rh_{13}$ core shell

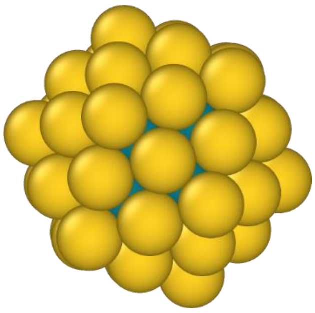

Rh	-0.0000624628	-0.0000795678	0.0005205913
Rh	-0.4615298583	1.0555872547	-2.3283620507
Rh	1.6770580006	1.8135743600	-0.8128624267
Rh	-1.6772704221	-1.8139029491	0.8122916122
Rh	0.4615091007	-1.0556043981	2.3287867738
Rh	-0.8558198743	2.4497442514	-0.0094856350
Rh	1.6044749369	-0.6772019742	-1.9299764078
Rh	-1.6046153783	0.6774617389	1.9301818196
Rh	0.8559485113	-2.4497536878	0.0092190865
Rh	-2.4883843701	0.3515877482	-0.6355985234
Rh	-0.9705918401	-1.5819401972	-1.8231238931
Rh	0.9710433191	1.5825418055	1.8227265387
Rh	2.4885176987	-0.3515100167	0.6355128181
Au	-0.9739367361	2.1907709856	-4.7912766067
Au	3.4688411068	3.7558495766	-1.6736103818
Au	-3.4686911540	-3.7556329221	1.6738608969
Au	0.9740224353	-2.1906908406	4.7910917979
Au	-1.7808958325	5.0238667975	-0.0296546287
Au	3.3298777767	-1.4107216440	-3.9821730273
Au	-3.3297623071	1.4106853832	3.9820692311
Au	1.7809118206	-5.0237980263	0.0297562610
Au	-5.0959768663	0.7411319072	-1.3191255722
Au	-2.0140980799	-3.2688333612	-3.7655042687
Au	2.0139601890	3.2686411887	3.7656838745
Au	5.0959971144	-0.7410862116	1.3191432357
Au	1.2939159616	3.0497898072	-3.3374882965

Au	-1.4021461237	3.7366932333	-2.4940571089
Au	0.8749937823	4.5254104785	-0.8722327541
Au	1.2138865312	0.4010774924	-4.5134901098
Au	3.4869697440	1.2087487851	-2.9132347394
Au	-3.1444082051	1.5015081636	-3.1670962502
Au	-3.5705045088	2.9907425640	-0.6879330157
Au	-1.5213671888	-0.5601804145	-4.4076291882
Au	0.6746363494	-2.3990754429	-3.9849218410
Au	-3.6809100231	-1.3142714492	-2.6172439147
Au	2.8149176192	3.6114176412	1.0748311317
Au	0.1239563133	4.2808145507	1.9266556256
Au	4.4228501571	1.5568262890	-0.1874136849
Au	4.3531679946	-1.0944497147	-1.3806562828
Au	3.6810066739	1.3143680757	2.6171286541
Au	-1.2938624483	-3.0496592616	3.3372862380
Au	-3.4868007035	-1.2086715556	2.9130598225
Au	-1.2138532781	-0.4009768329	4.5134276411
Au	-0.8749342649	-4.5253780005	0.8720524054
Au	1.4020966625	-3.7365766260	2.4939053179
Au	-4.4228515860	-1.5567761431	0.1872476871
Au	-4.3531303073	1.0944376203	1.3805666438
Au	-2.8147973517	-3.6113462258	-1.0750300848
Au	-0.1238478631	-4.2806547780	-1.9268297803
Au	1.5213765662	0.5603085301	4.4074061249
Au	-0.6745233630	2.3991370289	3.9847317420
Au	3.1444319823	-1.5013499335	3.1669846599
Au	3.5705164104	-2.9906695348	0.6879886264
Au	-2.6198162311	3.3283880527	2.0504753428
Au	2.6199638700	-3.3282796008	-2.0505517270

71-CM_ID

Rh	-1.1856773359	-1.5627289908	1.2931158365
Rh	-1.1291990039	2.7327061932	-1.5657147090
Rh	0.1899089685	-2.2119908502	-0.9481771089
Rh	-2.7637154106	1.8182230374	0.3313178744
Rh	1.0804540894	-0.4112907230	0.7096645920
Rh	1.9575284145	1.4128647347	2.3575939861
Rh	1.5481136896	0.0103152391	-1.8182874865
Rh	0.9441080343	2.0827283446	-0.0915610373
Rh	-3.1952390680	-0.8875741730	-0.2327225493
Rh	2.8571665706	-1.9405644644	-0.4208485851
Rh	3.2748828376	0.7497694485	0.0932190092
Rh	1.1997123643	-2.8823664983	1.5137667368
Rh	0.6183119410	-0.8220939138	3.1849162418
Rh	3.0942988401	-1.0576277701	2.1542533698
Rh	-0.9619132992	0.2718160796	-0.7260838795
Rh	-0.4665380000	0.7783224253	-3.1796355515
Rh	-1.8023797570	-1.4774963306	-2.4893218272
Rh	-0.7234876211	1.1401328716	1.8427466311
Rh	-2.9252209876	0.9394439638	-2.2281568753
Au	-0.7427853909	-4.2283516412	-2.7248095369
Au	2.0555093787	0.3387874165	-4.5830205309
Au	0.3768633942	2.4205682817	-5.2420556592
Au	1.5311887700	1.0717119702	5.1124688105
Au	4.3723510142	2.8047528446	1.8269942877
Au	-4.9971169733	1.9454018600	1.8352753417
Au	2.8611797412	3.3541712328	4.1147045292

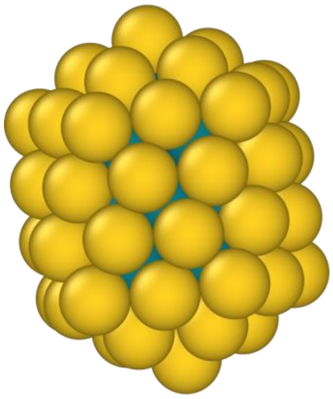

Au	1.3390733098	-5.5040272732	2.3440889956
Au	-3.1420843878	-0.0347781611	2.7039740779
Au	-1.8201469018	-2.1695624497	3.9943318248
Au	-2.1982852945	-3.6724653809	-0.3779393503
Au	5.5397891940	1.9961002301	-0.5936093567
Au	-4.4236485705	3.4876928585	-1.3332606042
Au	-1.1902631581	-4.2982061727	2.2010994136
Au	1.8417716955	4.1953172179	1.6212003793
Au	-5.3054179328	0.9744854630	-0.8175842493
Au	3.4702765149	-1.6638051908	-3.1188817180
Au	1.6623008657	2.7418054936	-2.8137395255
Au	0.7429220416	4.7863732219	-0.9802797322
Au	0.0970675548	3.1523034596	3.6966150402
Au	-3.6036273284	-2.8681071986	1.9824305833
Au	-0.4414491448	4.4983757581	-3.5304502390
Au	-4.9371672731	-2.9764913162	-0.4835037515
Au	5.2340430986	-1.7259885521	3.6937301799
Au	-2.1227366005	5.1901663603	-1.1209993639
Au	-5.3810864646	-0.8415030795	1.3240171985
Au	0.0981924870	-1.3020816064	5.7371694337
Au	-3.5499228168	-3.5778091398	-2.8720454616
Au	4.1779091965	0.8568988512	4.0466969546
Au	-4.8497258838	1.9122703865	-3.7088212783
Au	-3.1486514480	4.2650941271	-3.5781801092
Au	-0.9173220789	3.9456973443	1.1510960950
Au	-2.2949150212	2.0886677959	-4.9343762305
Au	0.7302014111	-3.5432431101	4.2269619505
Au	3.1279261586	-4.6112856429	0.3238711483
Au	-0.6203902825	-0.3178586729	-5.5510646115
Au	0.3083806635	-4.9858218333	-0.2140505213
Au	5.2581380424	-0.8430208284	-1.1239015518
Au	-1.2804930549	0.8048914970	4.6103362987
Au	-3.6948912789	4.3501921044	1.1477823479
Au	2.7531824514	-1.5659393867	4.9190867684
Au	5.0771255107	-2.7001573470	1.0223199231
Au	-2.6395223794	2.7669369281	3.1566918676
Au	3.2843444092	3.5659837329	-0.7253328714
Au	2.0849837989	-3.9516165250	-2.1903867245
Au	-4.8579209271	-1.1052424638	-2.5786240607
Au	3.3973582011	-3.7618711733	3.1240884061
Au	-1.7958112518	-2.7927549254	-4.8813437525
Au	5.5193417467	0.1684817096	1.6092076339
Au	0.7560402062	-2.0380545469	-3.7918056299
Au	-3.3281807152	-0.4472892136	-4.7277505448
Au	3.9749664366	1.1616160627	-2.7084671930

71-CM_DI

Rh	0.4753982337	-2.1723399882	-0.7021800327
Rh	-0.9442502301	1.3756554727	-2.8472001156
Rh	1.2477237581	0.1584790770	-1.9625056559
Rh	-2.3714475901	2.1979790160	-0.6746689810
Rh	1.0671008574	0.0145841498	0.6914238376
Rh	1.4243865631	2.1400419283	2.1123475898
Rh	0.2856245535	2.2649460096	-0.4934564194
Rh	-1.0510209137	1.2672989757	1.6456583675

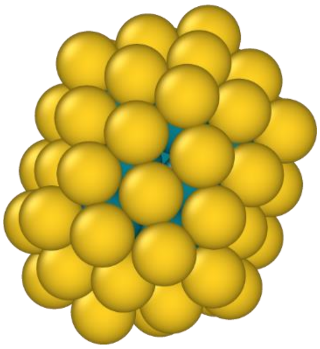

Rh	-3.1403057426	-0.0292083390	0.7335850324
Rh	3.0210461256	-1.1042860745	-0.4795767814
Rh	2.8291446724	1.6385429303	-0.1656495637
Rh	1.7610884280	-2.2862622063	1.6351763340
Rh	0.7003921068	-0.2834163550	3.1738272548
Rh	3.1753399412	0.0302346689	2.0799104279
Rh	-1.0600924941	-0.0123958612	-0.6979196238
Rh	-0.9216958790	-1.4221526388	-2.8223168347
Rh	-2.3348612207	-2.2515810420	-0.6453712009
Rh	-0.9489335942	-1.5021007522	1.5062331458
Rh	-3.1575823994	-0.0413997899	-2.1050925681
Au	-0.9515200687	-0.2183260751	-5.2818873551
Au	5.1686280311	1.8602972257	1.3692320709
Au	3.1177428979	2.3674001453	-2.8250844763
Au	-1.0769736050	-2.5105444628	4.1099386658
Au	5.2053707574	-2.0400291979	-1.7632034064
Au	-3.3727954751	1.9147702358	3.0464045192
Au	4.1168170216	-3.5513532415	0.3289346461
Au	5.1292320497	0.7611360085	-1.3471952097
Au	-1.1664915423	-4.7334677923	-0.3058762148
Au	2.7458632480	-3.1818048965	-2.3118709401
Au	-0.9790203065	-3.0738650356	-4.9930419506
Au	3.3375305451	4.0868093214	1.5643441319
Au	-2.3429022110	3.7918388522	-3.2032275322
Au	1.5988281806	-4.6717420675	-0.1628210121
Au	-2.3033506708	4.0000551030	1.4508477406
Au	0.6367210954	3.6482895285	-3.1349640978
Au	0.2096384392	-4.1291609564	-2.6956702678
Au	2.0341155618	4.4098984235	-0.8961723252
Au	-4.7484790667	2.5423569896	0.6783917899
Au	1.7499929870	4.2418512999	3.8757175660
Au	2.7444080705	-4.7407410470	2.4373191812
Au	-0.8866740781	4.7507937738	-1.0066432426
Au	-2.7047711037	-3.6438734545	1.9475827043
Au	5.4058073334	0.0545150978	3.5440962019
Au	-3.2931455876	-1.6433035491	-4.4453677841
Au	-2.5768077687	-3.9582224894	-2.8489938462
Au	-0.8507628486	3.1847440526	3.6774563006
Au	-3.9508165204	-4.4838520585	-0.4631812365
Au	1.1635304453	1.7441153901	5.0274013164
Au	-5.3782379878	-0.0372934960	-3.5900455797
Au	-3.2221717649	1.3773613251	-4.5836162000
Au	-5.3869177041	0.2398965836	2.1604409951
Au	-4.8570047527	-2.2020372496	0.8665936456
Au	-3.3162576457	-1.1924346726	3.2675197822
Au	5.4168255362	-1.0540783712	0.9036401392
Au	1.3775215398	-1.7524855069	-4.3026160962
Au	1.4162543288	1.1093591026	-4.5760527348
Au	-0.0272384883	-4.3119978127	2.2252051366
Au	-1.5391839240	0.5574412682	4.5536681885
Au	-4.6861857562	2.3820578288	-2.3075730687
Au	4.2207254743	-2.4580439806	3.0910671388
Au	0.4749029156	-0.9139731254	5.7439070573
Au	3.0419028361	-0.4404539829	4.8580721754
Au	0.4253563322	4.6215086700	1.4277144808
Au	3.5796092082	-0.4149998352	-3.3536934774
Au	-3.7176473229	4.5966963578	-0.9042356638
Au	3.7181096532	2.2623903761	3.8141643769

Au	-4.8131915360	-2.3424229008	-2.0600111305
Au	1.6879147267	-2.9788565105	4.3220527810
Au	-0.8410179730	2.6390821513	-5.2519745491
Au	-5.4891350986	0.1447695590	-0.7057655206
Au	4.6988464162	3.5212299175	-0.8373220267

Rh19-ID

Rh	-2.5478600060	-1.7717861905	0.7676556452
Rh	1.2017879344	0.0496464111	2.1888985580
Rh	-0.9044500712	1.4659686912	3.1554080838
Rh	-2.3430879656	-0.0817843267	4.7992135920
Rh	0.9564674396	-2.4443546855	3.2464407878
Rh	-1.3067067059	-3.5681787852	2.3639362049
Rh	-1.1798892365	-2.5236148377	4.8564383567
Rh	-1.0298727638	0.4325418378	0.6886336753
Rh	-4.6962752364	-2.7835106124	1.3531352814
Rh	-3.1881983843	0.3818603289	2.2721456368
Rh	-3.4821669610	-4.5393999987	2.9148132303
Rh	0.1210458247	-1.9843005819	0.7450804055
Rh	-1.0229733549	-0.9997825100	2.7415561362
Rh	0.3233502987	-0.3120320485	4.7359221621
Rh	-4.4961771218	-1.1295291070	5.2957342267
Rh	-5.5171924210	-3.1169571005	3.8581538470
Rh	-3.3582787779	-3.5174971408	5.3520618435
Rh	-3.2254065534	-2.0370817042	3.2884214406
Rh	-5.3221530876	-0.6764780296	2.8238747663

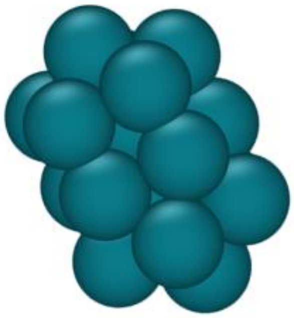

Rh19-DI

Rh	0.5114315022	-2.1678213356	-0.7333328310
Rh	-0.9367628481	1.4021343961	-2.7389653195
Rh	1.2911505029	0.1163199821	-1.9411734095
Rh	-2.3497846564	2.0551027723	-0.5810896415
Rh	1.0736170253	-0.0303576870	0.6534011423
Rh	1.4892313208	2.0754850545	1.9310093813
Rh	0.3374659805	2.1900186607	-0.4998930243
Rh	-1.0307893964	1.1870431771	1.5991284855
Rh	-3.1174274751	-0.1957782435	0.6085512239
Rh	3.0092941675	-1.2317208263	-0.3667284066
Rh	2.9041139111	1.4225278980	-0.2246903426
Rh	1.6610405489	-2.2190278474	1.7019155639
Rh	0.7226375191	-0.1761813466	3.1214935383
Rh	3.2132711778	-0.0187043030	2.0544850976
Rh	-0.9990584021	-0.0412616523	-0.7030299116
Rh	-0.8304408118	-1.2522249369	-2.8803385690
Rh	-2.1796036843	-2.2392539059	-0.8118479106
Rh	-0.9233678563	-1.5061721670	1.4542857367
Rh	-3.1388485257	-0.0519876892	-2.1036308031

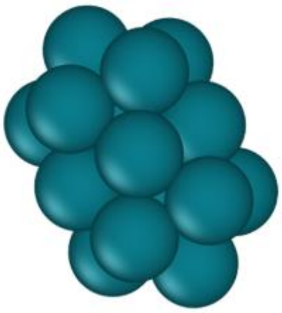

Rh_{55} icosahedron

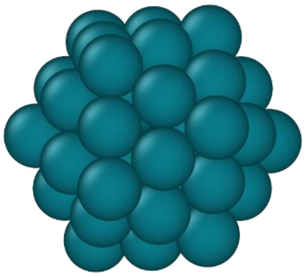

Rh	-0.3961597083	2.1526316220	-5.8765840552
Rh	-0.2008448205	1.0891184984	-3.6083568699
Rh	-0.0000109802	-0.0000609506	-1.2831098580
Rh	-0.4205947408	1.5316079874	0.7450366699
Rh	-0.2217456707	0.4467523237	3.1105256751
Rh	2.0172381525	1.4763953278	-4.9341894458
Rh	0.6206589253	3.5568716882	-3.8399087023
Rh	-2.2848149241	0.4454550471	-5.0462156624
Rh	-1.6582869448	-2.2001034176	-4.7465959135
Rh	2.6440608422	-1.1689750491	-4.6342157259
Rh	2.1979050845	0.3732687341	-2.5738681032
Rh	0.8158354602	2.4333328495	-1.4894716230
Rh	-2.0620474270	-0.6474817135	-2.6852127419
Rh	-0.8158396352	-2.4334238466	-1.0766910475
Rh	0.4205849183	-1.5317004500	-3.3112571084
Rh	1.8192234788	-1.8033441059	-1.0080057658
Rh	2.0619966856	0.6474213363	0.1190267676
Rh	-1.8192473591	1.8032745551	-1.5581599629
Rh	-2.1979098795	-0.3733026612	0.0076869658
Rh	0.2008427265	-1.0891887931	1.0421839527
Rh	2.2847975320	-0.4454312870	2.4801283223
Rh	1.6582758418	2.2000898114	2.1804601517
Rh	-0.8303804140	3.0264124505	2.7246330837
Rh	-2.6440468735	1.1689931319	2.0680797629
Rh	0.3962070184	-2.1525354946	3.3105194706
Rh	4.3425076144	0.7391183703	-3.8333505945
Rh	3.0429771464	2.8351627241	-2.7944113189
Rh	1.6124567825	4.8061125372	-1.6914096151
Rh	-1.0130420046	4.2763379648	-1.7690268661
Rh	-2.0392893225	2.9204198356	-3.9095207703
Rh	-4.0746849865	-1.2797094841	-4.0529851288
Rh	-2.9055729818	-3.1115413983	-2.4902865053
Rh	-0.3987118852	-4.0029070805	-3.1226651638
Rh	0.2217788536	-0.4467351712	-5.6766667393
Rh	0.8304228973	-3.0264541347	-5.2908260169
Rh	4.0550226820	-1.4436466496	-2.3081233832
Rh	4.2998272218	1.0307706878	-1.1706947385
Rh	2.9055823068	3.1115630559	-0.0758117725
Rh	-3.5937026158	3.5642171609	-1.8267732631
Rh	-3.9183302660	1.1674150489	-2.9758345019
Rh	-1.6124257867	-4.8061600709	-0.8745784900
Rh	-3.0429087243	-2.8351103507	0.2283312654
Rh	-0.6206128031	-3.5569033108	1.2738550473
Rh	1.0130548024	-4.2763671088	-0.7969827802
Rh	2.2609432565	-3.3673339588	-3.0537216423
Rh	3.9182445215	-1.1673941009	0.4097588094
Rh	4.0745750975	1.2797855746	1.4868778481
Rh	0.3987245933	4.0028674332	0.5565608146
Rh	-2.2609590533	3.3673182378	0.4876222648
Rh	-4.0550661679	1.4436978508	-0.2579587693
Rh	-4.2998528244	-1.0306771036	-1.3954077540
Rh	-4.3424645409	-0.7389689123	1.2673025132
Rh	-2.0171986847	-1.4763411761	2.3680764704
Rh	2.0393080692	-2.9204325777	1.3434705219
Rh	3.5937085146	-3.5641814867	-0.7392279799

Au_{20} tetrahedron

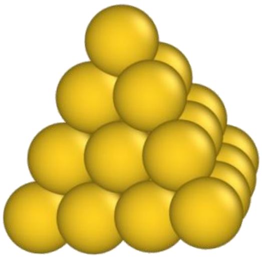

Au	-0.3538646439	1.4604957026	-2.9364494170
Au	1.7550881450	-0.1588270046	-2.7886923604
Au	3.8457349984	-1.8348898080	-2.4758178288
Au	-2.5273915201	3.0596314400	-2.9228717062
Au	-0.6298069375	-0.9624572491	-1.5453654349
Au	-2.8284308137	0.7827259869	-1.5071296227
Au	1.6065075317	-2.6228534092	-1.1946424002
Au	1.1466470950	1.4415873698	-0.5643659693
Au	-2.9330079852	-3.6870082282	1.4456678497
Au	-1.1703003893	3.0274043836	-0.5911463937
Au	3.2657390227	-0.3780401841	-0.2794906197
Au	-1.5038022952	0.7165991742	0.9675221953
Au	0.9876402518	-1.1959478731	1.1427272859
Au	-2.9622304196	-1.4497862942	-0.0618896170
Au	-0.6367343303	-3.2356570189	0.1024607240
Au	0.1992718916	2.8301654585	1.6836878850
Au	2.5268399210	1.0437012736	1.8474031023
Au	-1.4522659523	-1.6675050186	2.4491188034
Au	0.0517630672	0.3675968587	3.2783821674
Au	1.6131833627	2.4634344402	3.9511713568

Au_{55} icosahedron

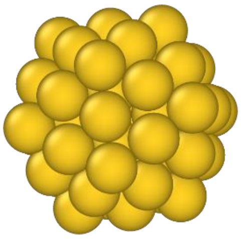

Au	-0.4263282068	2.3148325137	-6.1908772922
Au	-0.2159862509	1.1662502959	-3.7724718959
Au	-0.0000113977	-0.0000117903	-1.2830518396
Au	-0.4530443350	1.6441341579	0.8923365034
Au	-0.2423200253	0.4872857995	3.4891496967
Au	2.2015608564	1.6120433779	-5.2652784784
Au	0.6775495142	3.8806003785	-4.0749562533
Au	-2.4939284574	0.4857597121	-5.3862670852
Au	-1.8094417426	-2.4010794258	-5.0614648056
Au	2.8855750567	-1.2764142313	-4.9394497441
Au	2.3572323785	0.3992126259	-2.6680344009
Au	0.8708978624	2.5959646767	-1.5027981015
Au	-2.2125574735	-0.6942760539	-2.7887773369
Au	-0.8709523757	-2.5959536911	-1.0633047706
Au	0.4529889037	-1.6441801974	-3.4584466846
Au	1.9551396901	-1.9330298621	-0.9879284142
Au	2.2125123000	0.6942822954	0.2227217588
Au	-1.9551705400	1.9329809997	-1.5781343867
Au	-2.3572561355	-0.3992114570	0.1018836201
Au	0.2159336084	-1.1662421823	1.2063843722
Au	2.4939041597	-0.4857527303	2.8202349129
Au	1.8093917168	2.4010553034	2.4954298172
Au	-0.8926883991	3.2499481026	3.0133488046
Au	-2.8855445347	1.2763933863	2.3732172088
Au	0.4263236471	-2.3147817388	3.6247744729
Au	4.6507485557	0.7934311886	-4.0225950180
Au	3.3253609301	3.0903428973	-2.9367118815
Au	1.7373615398	5.1136109842	-1.7229591536
Au	-1.1059903144	4.6469425072	-1.8142145419
Au	-2.2284674094	3.1879534343	-4.1481823858

Au	-4.3681356181	-1.3744314970	-4.2575195089
Au	-3.1740092934	-3.3930715326	-2.6051242367
Au	-0.4349623354	-4.3595496560	-3.2920582181
Au	0.2422603167	-0.4872894283	-6.0552661540
Au	0.8926898359	-3.2499830110	-5.5794893455
Au	4.4180930087	-1.5755579785	-2.4019329117
Au	4.6769190696	1.1259920592	-1.1605902245
Au	3.1739826313	3.3931421307	0.0391011713
Au	-3.8577226807	3.8231808979	-1.8670601532
Au	-4.2706280317	1.2738927847	-3.1311012204
Au	-1.7373160686	-5.1136113985	-0.8432499715
Au	-3.3252571040	-3.0903019459	0.3705792570
Au	-0.6775640095	-3.8805570094	1.5087988237
Au	1.1059955946	-4.6469683861	-0.7519324868
Au	2.4682582707	-3.6736325007	-3.2152450430
Au	4.2706366877	-1.2739219252	0.5651245025
Au	4.3681142886	1.3744625750	1.6914881468
Au	0.4349668184	4.3595201014	0.7258952133
Au	-2.4681850228	3.6735972118	0.6490863867
Au	-4.4180225360	1.5755712861	-0.1641560694
Au	-4.6768921146	-1.1259241237	-1.4054680778
Au	-4.6506961282	-0.7934218308	1.4564519396
Au	-2.2015265659	-1.6120166453	2.6990765232
Au	2.2284314677	-3.1880152179	1.5820968399
Au	3.8577854012	-3.8231962366	-0.6990518813
